# Briarenols Q–T: Briaranes from A Cultured Octocoral *Briareum stechei* (Kükenthal, 1908)

**DOI:** 10.3390/md18080383

**Published:** 2020-07-24

**Authors:** Yi-Lin Zhang, Chih-Chao Chiang, Yi-Ting Lee, Zhi-Hong Wen, Yang-Chang Wu, Yu-Jen Wu, Tsong-Long Hwang, Tung-Ying Wu, Chia-Yuan Chang, Ping-Jyun Sung

**Affiliations:** 1 Graduate Institute of Marine Biology, National Dong Hwa University, Pingtung 944401, Taiwan; 610863012@gms.ndhu.edu.tw; 2 National Museum of Marine Biology and Aquarium, Pingtung 944401, Taiwan; 3 Graduate Institute of Clinical Medical Sciences, College of Medicine, Chang Gung University, Taoyuan 333323, Taiwan; moonlight0604@hotmail.com; 4 Department of Marine Biotechnology and Resources, National Sun Yat-sen University, Kaohsiung 804201, Taiwan; etigh20252qq@gmail.com (Y.-T.L.); wzh@mail.nsysu.edu.tw (Z.-H.W.); 5 Institute of BioPharmaceutical Sciences, National Sun Yat-sen University, Kaohsiung 804201, Taiwan; 6 Graduate Institute of Integrated Medicine, College of Chinese Medicine, China Medical University, Taichung 404333, Taiwan; yachwu@mail.cmu.edu.tw; 7 Chinese Medicine Research and Development Center, China Medical University Hospital, Taichung 404394, Taiwan; 8 Department of Nursing, Meiho University, Pingtung 912009, Taiwan; x00002180@meiho.edu.tw; 9 Research Center for Chinese Herbal Medicine, Research Center for Food and Cosmetic Safety, Graduate Institute of Healthy Industry Technology, College of Human Ecology, Chang Gung University of Science and Technology, Taoyuan 333324, Taiwan; htl@mail.cgu.edu.tw; 10 Graduate Institute of Natural Products, College of Medicine, Chang Gung University, Taoyuan 333323, Taiwan; 11 Chinese Herbal Medicine Research Team, Healthy Aging Research Center, Chang Gung University, Taoyuan 333323, Taiwan; 12 Department of Anaesthesiology, Chang Gung Memorial Hospital, Taoyuan 333423, Taiwan; 13 Department of Chemical Engineering, Ming Chi University of Technology, New Taipei City 243303, Taiwan; 14 Department of Biological Science & Technology, Meiho University, Pingtung 912009, Taiwan; 15 Department of Food Science and Nutrition, Meiho University, Pingtung 912009, Taiwan; 16 Department of Neurosurgery, Antai Medical Care Corporation Antai Tian-Sheng Memorial Hospital, Pingtung 928004, Taiwan; 17 Graduate Institute of Natural Products, Kaohsiung Medical University, Kaohsiung 807378, Taiwan

**Keywords:** *Briareum stechei*, briarane, briarenol, iNOS, COX-2

## Abstract

Our continuous chemical study of a cultured octocoral *Briareum stechei* led to the isolation of four new briarane diterpenoids, briarenols Q–T (**1**–**4**). The structures of new metabolites **1**–**4** were established by spectroscopic methods, and compounds **3** and **4** were found to inhibit the generation of inducible nitric oxide synthase (iNOS) from RAW 264.7 stimulated by lipopolysaccharides (LPS).

## 1. Introduction

**Figure 1:**
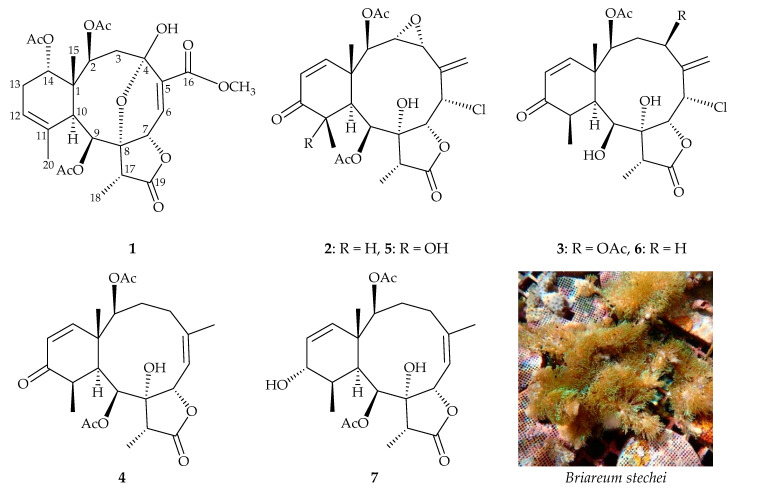
Structures of briarenols Q–T (**1**–**4**), pachyclavulide D (**5**), solenolide E (**6**), cavernulin B (**7**), and a picture of cultured *Briareum stechei*.

Since 1950, the nucleoside spongothymidine, which is obtained from the Caribbean sponge *Cryptotethia* [1], and the derivative of this nucleoside 1-β-D-arabinofuranosylcytosine (Ara-C) have been approved as the first marine-origin clinical medications used in treatment of leukemia in 1969. Marine natural products (MNPs) from marine invertebrates, such as Porifera and Cnidaria, played important roles in drug discovery due to their complex structures and interesting bioactivities [2,3]. Most of the pharmaceutical coral reef organisms are claimed to be endangered species. In order to protect natural populations and habitats of these marine organisms from overexploitation and to support bioactive materials for further study and medicinal use [4], a cultured octocoral *Briareum stechei* (Kükenthal, 1908), which was previously identified as *Briareum excavatum* (Nutting, 1911) [5], was studied for its interesting chemical constituents related to briarane-type diterpenoids. Herein, we report on the isolation, structure determination, and anti-inflammatory activity of four unreported isolates, briarenols Q–T (**1**–**4**) (Figure 1).

## 2. Results and Discussion

The octocoral *B. stechei* was harvested in an 80 ton culturing tank in April 2016. This target organism was freeze-dried and ground, followed by exhaustive extraction in a mixture of dichloromethane (CH_2_Cl_2_) and methanol (MeOH). Normal- and reverse-phase HPLC yielded four new briarane diterpenoids, briarenols Q–T (**1**–**4**).

Briarenol Q (**1**), ${\left[\alpha \right]}_{D}^{25}$ +22 (*c* 0.1, CHCl_3_), was obtained as an amorphous powder with the molecular formula C_27_H_34_O_12_, based on its positive-mode high-resolution electrospray ionization mass spectrum (HRESIMS) at *m/z* 573.19426 (calculated for C_27_H_34_O_12_Na^+^, 573.19425), with 11 indices of hydrogen deficiency (IHDs). The IR spectrum showed the presence of hydroxy (ν_max_ 3408 cm**^–^**^1^), γ-lactone (ν_max_ 1783 cm**^–^**^1^), and ester carbonyl (ν_max_ 1733 cm**^–^**^1^) groups. The ^1^H, ^13^C, and heteronuclear single quantum coherence (HSQC) spectra indicated the presence of seven methyls, two sp^3^ methylenes, six sp^3^ methines, three sp^3^ quaternary carbons, two sp^2^ methines, two quaternary olefinic carbons, and five ester-equivalents (Table 1 and Table 2), accounting for all carbons and 33 of the 34 protons. Two of the sp^3^ quaternary carbons had ^13^C chemical shifts at δ_C_ 80.8 and 94.0, indicative of an oxygen-bearing quaternary carbon and a hemiketal carbon, respectively. A tetracyclic scaffold was deduced by IHDs, as seven of the 11 unsaturation degrees could be assigned to a pair of carbon–carbon double bonds (δ_C_ 138.4, C-5; 128.0, CH-6; 131.1, C-11; 122.0, CH-12) and five esters (δ_C_ 164.6, 169.2, 169.7, 173.2, 174.9).

**Figure 2:**
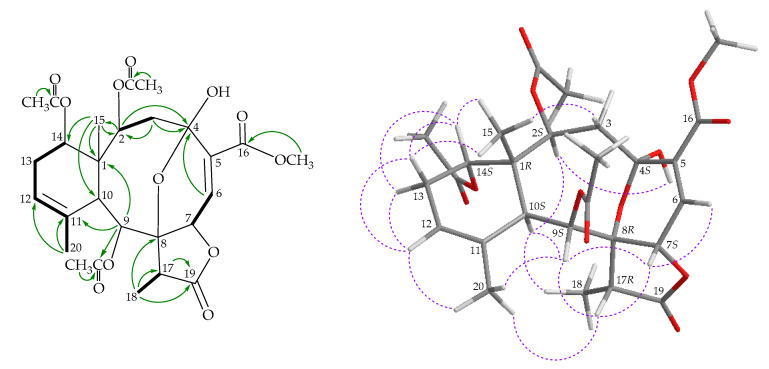
Key correlation spectroscopy (COSY; 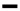), heteronuclear multiple-bond correlation (HMBC; 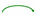), and nuclear Overhauser effect spectroscopy (NOESY; 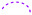) correlations of **1**.

The ^1^H NMR coupling information in the ^1^H–^1^H correlation spectroscopy (COSY) of **1** enabled the identification of four different spin systems, H-2/H_2_-3, H-6/H-7, H-12/H_2_-13/H-14, and H-17/H_3_-18 (Figure 2), which were assembled with the assistance of a heteronuclear multiple-bond correlation (HMBC) experiment. The ^2^*J*- or ^3^*J*-^1^H–^13^C long-range correlations between H-2, H-3α, H-6/C-4, H-3α/C-2, H-9/C-1, and H-9/C-11 permitted elucidation of the main carbon skeleton of **1**. A vinyl methyl at C-11 was confirmed by an allylic coupling between H-12 (δ_H_ 5.56) and H_3_-20 (δ_H_ 1.96) in the COSY experiment. The ^2^*J*- or ^3^*J*-^1^H–^13^C long-range correlations between H-2, H-3α, H-6/C-4, H-3α/C-2, H-9/C-1, and H-9/C-11 permitted elucidation of the main carbon skeleton of **1**. A vinyl methyl at C-11 was confirmed by an allylic coupling between H-12 (δ_H_ 5.56) and H_3_-20 (δ_H_ 1.96) in the COSY experiment and by the HMBC between H_3_-20/C-11 and C-12. The ring junction Me-15 at C-1 was supported by the HMBC between H_3_-15/C-1, C-2, C-10, C-14, and H-2/C-15. Thus, the methyl-esterified carboxyl group at C-5 was supported by an HMBC between δ_H_ 3.81 (3H, s, –OMe), with δ_C_ 164.6 (C-16). The acetate ester at C-9 was established by a correlation between H-9 (δ_H_ 6.05) and the acetate carbonyl at δ_C_ 169.7 observed in the HMBC spectrum. The C-4 hydroxy group was concluded to be a part of a hemiketal constellation on the basis of a characteristic carbon signal at δ_C_ 94.0 (a quaternary hemiketal carbon, C-4). An oxygenated quaternary carbon signal at δ_C_ 80.8 showed a ^3^*J*-coupling with the methyl protons at δ_H_ 1.46 (H_3_-18). In total, 8 of the 12 oxygen atoms in the molecular formula could be accounted for the presence of a γ-lactone, an ester, a hemiketal, and an α,β-unsaturated methyl esterified carboxyl group. Thus, the remaining four oxygen atoms had to be positioned at C-2 and C-14 as acetoxy groups, respectively, as indicated by their ^1^H and ^13^C NMR chemical shifts (δ_H_ 5.11, 1H, d, *J* = 7.2 Hz; δ_C_ 72.1, CH-2; δ_H_ 5.18, 1H, d, *J* = 4.2 Hz; δ_C_ 71.8, CH-14), although no HMBC correlation was observed from H-2 and H-14 to any acetate carbonyl. These findings, together with the HMBC between H-17/C-19 and H_3_-18/C-17 and C-19, were used to establish the molecular framework of **1**.

The stereochemical evaluation of **1** was approached using a nuclear Overhauser effect spectroscopy (NOESY) experiment (Figure 2) and was found to be compatible with that of **1** offered by computer modeling [6] and that obtained from vicinal proton coupling constant analysis. Proton H-10 exhibited correlations with H-2, H-9, and H_3_-18, while H-14 correlated with H_3_-15, setting the Me-15 at C-1 as *trans* to H-10, as observed in all naturally occurring briarane-type diterpenoids [7]. Due to Me-18 at C-17 being α-oriented in the γ-lactone moiety, H-17 should be positioned on the β face. This proton showed a slight correlation with H-7, indicating that H-7 was β-oriented. H-7 showed a correlation with H-6, and a coupling constant (*J* = 4.8 Hz) was detected between H-7 and H-6, indicating that the dihedral angle between H-6 and H-7 is approximately 60°, and that H-6 is β-oriented. The hydroxy proton at δ_H_ 6.11 (OH-4) displayed a light correlation with H-2, setting the hydroxy group at C-4 in an S*-configuration. The NOESY spectrum also showed correlations of H-6/ H-7 and OH-4/H-2 and with H-12/H_3_-20, revealing the *E*-geometry and *Z*-geometry of C-5/C-6 and C-11/C-12 double bonds, respectively. The remaining stereogenic carbon, C-8, lacked a proton, but there were correlations between H-7/H-17 and H-7/H-9, indicating that C-8 was in an R*-configuration, as evidenced by modeling analysis. Based on the above findings, the relative configurations of stereogenic carbons of **1** were elucidated as 1R*,2S*,4S*,7S*,8R*,9S*,10S*,14S*, and 17R*. As briaranes **1**–**4** were isolated along with the known briaranes excavatolide A and briaexcavatolide F [8,9] from the same target organism, the absolute configurations of these two compounds were determined by single-crystal X-ray diffraction analysis [10]. Therefore, it is reasonable on biogenetic grounds to conclude that briaranes **1**–**4** have the same absolute configuration as those of excavatolide A and briaexcavatolide F. Based on the above findings, the configurations of the stereogenic carbon of **1** were elucidated as 1*R*,2*S*,4*S*,7*S*,8*R*,9*S*,10*S*,14*S*, and 17*R* (Supplementary Materials, Figures S1–S10).

**Figure 3:**
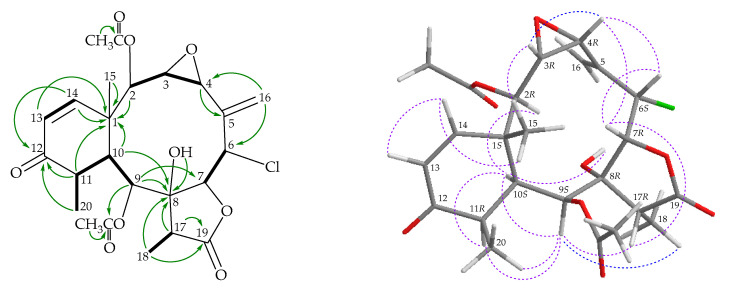
Key COSY (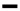), HMBC (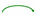), and NOESY (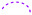) correlations of **2**.

Briarane **2** (briarenol R) was isolated as an amorphous powder that showed two sodiated adduct ion peaks in (+)-HRESIMS at *m/z* 519.13912 and 521.13596 (3:1), which accounted for a chlorine atom in the molecular formula, C_24_H_29_^35^ClO_9_ (calculated for C_24_H_29_^35^ClO_9_ + Na, 519.13923) (10 degrees of unsaturation). The IR spectrum of **2** showed α,β-unsaturated ketone, ester carbonyl, γ-lactone, and broad OH stretching at 1682, 1742, 1783, and 3455 cm^–1^, respectively. From the ^13^C and ^1^H NMR (Table 1 and Table 2), HSQC, and HMBC spectra (Figure 3), an α,β-unsaturated ketone was deduced from the signals of three carbons at δ_C_ 201.6 (C-12), 124.4 (CH-13), and 152.5 (CH-14). The presence of an exocyclic olefin was confirmed by the signal of an sp^2^ methylene carbon at δ_C_ 120.3 (CH_2_-16) and further supported by two olefin proton signals at δ_H_ 5.79 (1H, d, *J* = 3.0 Hz, H-16a) and 6.06 (1H, d, *J* = 3.0 Hz, H-16b) in the ^1^H NMR spectrum. In addition, three carbonyl resonances at δ_C_ 173.7 (C-19), 169.6, and 169.3 (2 × ester carbonyls) confirmed the presence of γ-lactone and two ester groups; two acetate methyls (δ_H_ 2.23 and 2.27, both 3H × s; δ_C_ 20.9 and 21.8, CH_3_ × 2) were observed. A disubstituted epoxy group was confirmed from the signals of two oxymethine carbons at δ_C_ 60.2 (CH-3) and 57.4 (CH-4). The chemical shifts of oxymethine protons at δ_H_ 3.48 (1H, dd, *J* = 9.0, 4.2 Hz, H-3) and 3.67 (1H, d, *J* = 4.2 Hz, H-4) further confirmed the presence of this group. Based on the ^13^C NMR data and unsaturated numbers, **2** was established as a tetracyclic briarane. It was observed that the spectroscopic data of **2** resembled those of a known briarane, pachyclavulide D (**5**) (Figure 1), obtained from the soft coral *Pachyclavularia violacea* [11]. Comparison of the proton chemical shifts, coupling patterns, and coupling constants for oxymethine protons H-3 (δ_H_ 3.38, 1H, dd, *J* = 9.0, 4.2 Hz) and H-4 (δ_H_ 3.67, 1H, d, *J* = 4.2 Hz) of **2** with those of **5** (δ_H_ 3.49, 1H, dd, *J* = 8.9, 3.7 Hz, H-3; 3.66, 1H, d, *J* = 3.7 Hz, H-4) [11] indicated the 3,4-epoxide group towards the α-side of the briarane system as that of **5**. Furthermore, the 1D and 2D NMR spectra revealed that the signals corresponding to the 11α-hydroxy group in **5** were replaced by those of a proton in **2**. Therefore, briarenol R (**2**) was assigned as having a structure with the same stereochemistry as **5** because of the stereogenic carbons that **2** has in common with **5**, as confirmed by correlations observed in the NOESY spectrum (Figure 3). Therefore, the configurations of the stereogenic carbons of **2** were elucidated as 1*S*,2*R*,3*R*,4*R*,6*S*, 7*R*,8*R*,9*S*,10*S*,11*R*, and 17*R* (Supplementary Materials, Figures S11–S20).

**Figure 4:**
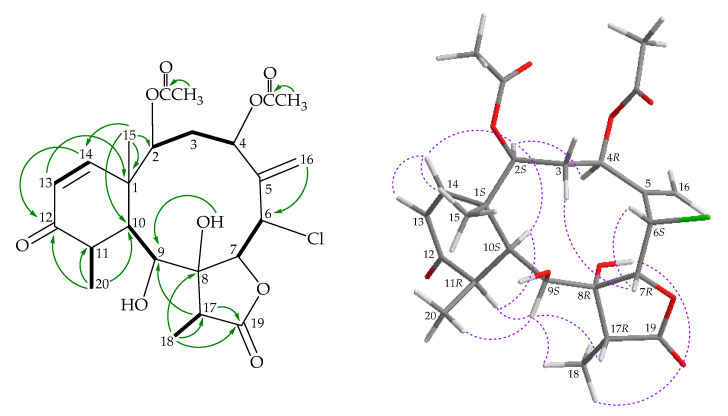
Key COSY (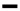), HMBC (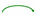), and NOESY (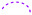) correlations of **3**.

The molecular formula of briarenol S (**3**), containing a chlorine atom, was found to be C_24_H_31_ClO_9_ by (+)-HRESIMS at *m/z* 521.15499 (calculated for C_24_H_31_^35^ClO_9_, 521.15488). The IR spectrum showed absorptions at 3459 cm^–1^ due to hydroxy groups at 1777, 1737, and 1678 cm^–^^1^, due to γ-lactone, ester carbonyl, and α,β-unsaturated ketonic carbonyl groups, respectively. The ^13^C and ^1^H NMR spectra (Table 1 and Table 2) disclosed the signals due to five methyls, an sp^3^ methylene, eight sp^3^ methines, two sp^3^ quaternary carbons, an sp^2^ methylene, two sp^2^ methines, an sp^2^ quaternary carbon, and four carbonyl carbons. The low-field ester carbonyl carbon (δ_C_ 175.6), coupled with the IR absorption at 1777 cm^–1^, suggested the presence of a γ-lactone moiety in **3**. The high-field ketonic carbonyl carbon at δ_C_ 202.4 suggested that the carbonyl group was conjugated with a carbon–carbon double bond. The ^1^H NMR spectrum (Table 2) showed the signals due to two secondary methyls, a tertiary metyl, two acetate methyls, an aliphatic methylene, three aliphatic methines, a methine bearing a chlorine atom, four oxymethines, an exomethylene, and two olefinic methines. These spectroscopic data, coupled with the degrees of unsaturation (IHDs = 9), suggested that compound **3** is a tricyclic diterpenoid with a γ-lactone, an α,β-unsaturated ketone, an exocyclic olefin, and two acetoxy groups. It was found that the spectroscopic data of **3** were similar to those of a known briarane, solenolide E (**6**), isolated from a soft coral identified as *Solenopodium* sp. (= *Briareum* sp.) [5], collected in the Western Caroline Islands of Palau [12], except that the signals corresponding to one of the C-4 methylene protons in **6** were replaced by signals for an acetoxy group in **3**. In the NOESY experiment, H-4 showed a correlation with H-2, revealing that the acetoxy group at C-4 is on the β face in **3**. The HMBC and COSY correlations, as shown in Figure 4, provided the gross structure for **3**. Hence, briarenol S (**3**) was found to be the 4β-acetoxy derivative of **6** and the stereochemistry of the stereogenic carbons in **3** was deduced by NOESY analysis (Figure 4), determined as 1*S*,2*S*,4*R*,6*S*,7*R*, 8*R*,9*S*,10*S*,11*R*, and 17*R* (Supplementary Materials, Figures S21–S31).

**Figure 5:**
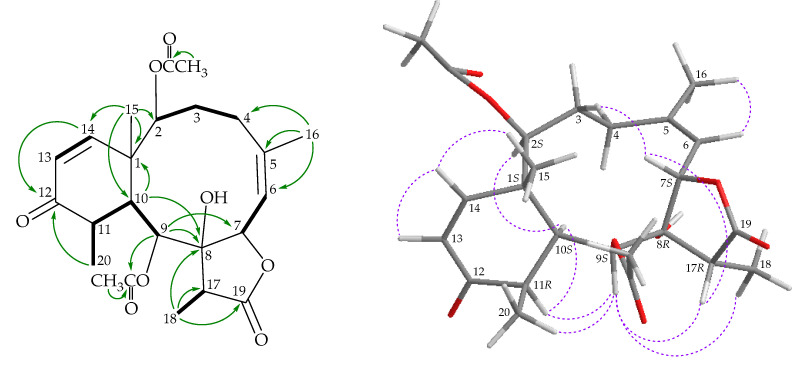
Key COSY (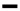), HMBC (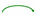), and NOESY (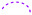) correlations of **4**.

Our present study has led to the isolation of a new briarane, briarenol T (**4**). Its molecular formula C_24_H_32_O_8_ was deduced from (+)-HRESIMS at *m/z* 471.19879 (calculated for C_24_H_32_O_8_ + Na, 471.19894). The IR spectrum showed absorptions that indicated three different carbonyl types: γ-lactones (ν_max_ 1772 cm^−1^), esters (ν_max_ 1740 cm^–1^), and α,β-unsaturated ketones (ν_max_ 1683 cm^–1^). The latter structural feature was confirmed by the presence of signals at δ_C_ 202.6 (C-12), 154.6 (CH-14), and 124.1 (CH-13) in the ^13^C NMR spectrum (Table 1), and the presence of a mutually coupled pair of doublet signals in the ^1^H NMR spectrum at δ_H_ 5.85 (H-13) and 6.39 (H-14) (*J* = 10.2 Hz) corresponding to the α- and β-olefinic protons, respectively (Table 2). The spectroscopic data of **4** were similar to those of a known diterpene, cavernulin B (**7**) (Figure 1), isolated from a sea pen, *Cavernularia* sp., collected from the Eastern Coast of Bay of Bengal near Digna, India [13], except that the signals corresponding to the 12-hydroxy group in **7** disappeared and were replaced by a ketone group in **4**, as assessed by comparing the related spectroscopic data of **4** with those of **7**. The locations of functional groups were confirmed by 2D NMR correlations (Figure 5), and hence the structure of briarenol T was assigned as **4**, and the configurations of the stereogenic carbons were elucidated as 1*S*,2*S*,7*S*,8*R*,9*S*,10*S*,11*R*, and 17*R* (Figure 4) (Supplementary Materials, Figures S32–S41).

The effects of briaranes **1**–**4** on the release of iNOS and COX-2 from LPS-stimulated RAW 264.7 macrophage cells were assessed (Table 3). Briaranes **3** and **4** were found to inhibit the release of iNOS to 78.50 and 79.95%, respectively. It is interesting to note that **2** enhanced the expression of COX-2 to 112.96%, as compared to results of the cells stimulated with LPS only (Supplementary Materials, Figure S37).

## 3. Materials and Methods

### 3.1. General Experimental Procedures

NMR spectra were recorded on a 600 MHz Jeol ECZ NMR (Jeol, Tokyo, Japan) spectrometer using the residual CHCl_3_ (δ_H_ 7.26 ppm) and CDCl_3_ (δ_C_ 77.1 ppm) signals as internal references for ^1^H and ^13^C NMR, respectively. ESIMS and HRESIMS were obtained from the Bruker mass spectrometer with 7 Tesla magnets (model: SolariX FTMS system, Bremen, Germany). Column chromatography, HPLC, IR spectra, and optical rotation were performed according to our earlier research [14].

### 3.2. Animal Material

Specimens of *B. stechei* used for this study were collected from an 80 ton culturing tank equipped with a flow-through sea water system located in the National Museum of Marine Biology and Aquarium (NMMBA) at April 2016. Identification of the species of this organism was performed by comparison, as described in previous studies [5,15,16,17]. Living reference specimens are being maintained in the authors’ marine organisms culturing tanks and a voucher specimen was deposited in the NMMBA (voucher no.: NMMBA-TW-GC-2016-031), Taiwan.

### 3.3. Extraction and Isolation

Sliced bodies (wet/dry weight = 3980/1860 g) of the specimen were grounded and extracted with a mixture of MeOH and CH_2_Cl_2_ (1:1) to give an extract (104 g). The extract was then applied to a silica gel column chromatograph (Si C.C.) and eluted with gradients of hexane/EtOAc (stepwise from 50:1−1:2) to furnish fractions A−L. Fractions H and I were combined (19.0 g) and separated on Si C.C. using hexane/EtOAc (stepwise from 50:1, pure EtOAc) to obtain fractions H1−H8. Fraction H6 was chromatographed with Si C.C. using hexane/EtOAc/acetone to obtain fractions H6A−H6K. Fraction H6E was separated by Si C.C. using a mixture of CH_2_Cl_2_ and acetone (4:1) to obtain fractions H6E1−H6E6. Fraction H6E2 was repurified by NP-HPLC using a mixture of CH_2_Cl_2_ and acetone (8:1; at a flow rate = 2.0 mL/min) to yield fractions H6E2A−H6E2E. Fraction H6E2C was separated by RP-HPLC using a mixture of MeOH and H_2_O (60:40; at a flow rate = 4.0 mL/min) to obtain **2** (0.6 mg), **4** (0.3 mg), and **1** (0.3 mg), respectively. Fraction H6G was chromatographed on reverse-phase silica gel, using a mixture of acetonitrile and H_2_O (1:1) to obtain fractions H6G1–H6G5. Fraction H6G3 was repurified by NP-HPLC using a mixture of dichloromethane and acetone (5:1) to yield fractions H6G3A−H6G3D. Fraction H6G3B was separated by NP-HPLC using a mixture of dichloromethane and acetone (8:1) to yield fractions H6G3B1−H6G3B5. Fraction H6G3B5 was separated by RP-HPLC with a mixture of MeOH and H_2_O (60:40, at a flow rate of 1.0 mL/min) to afford **3** (0.3 mg).

Briarenol Q (**1**): Amorphous powder; ${\left[\alpha \right]}_{D}^{25}$ +22 (*c* 0.1, CHCl_3_); IR (KBr) ν_max_ 3408, 1783, 1733 cm^−1^; ^13^C (150 MHz, CDCl_3_) and ^1^H (600 MHz, CDCl_3_) NMR data, see Table 1 and Table 2; ESIMS: *m/z* 573 [M + Na]^+^; HRESIMS: *m/z* 573.19426 (calculated for C_27_H_34_O_12_ + Na, 573.19425).

Briarenol R (**2**): Amorphous powder; ${\left[\alpha \right]}_{D}^{22}$ −55 (*c* 0.2, CHCl_3_); IR (KBr) ν_max_ 3455, 1783, 1742, 1682 cm^−1^; ^13^C (150 MHz, CDCl_3_) and ^1^H (600 MHz, CDCl_3_) NMR data, see Table 1 and Table 2; ESIMS: *m/z* 519 [M + Na]^+^, 521 [M + 2 + Na]^+^; HRESIMS: *m/z* 519.13912 (calculated for C_24_H_29_^35^ClO_9_ + Na, 519.13923).

Briarenol S (**3**): Amorphous powder; ${\left[\alpha \right]}_{D}^{24}$ +34 (*c* 0.1, CHCl_3_); IR (KBr) ν_max_ 3459, 1777, 1738, 1678 cm^−1^; ^13^C (150 MHz, CDCl_3_) and ^1^H (600 MHz, CDCl_3_) NMR data, see Table 1 and Table 2; ESIMS: *m/z* 521 [M + Na]^+^, 523 [M + 2 + Na]^+^; HRESIMS: *m/z* 521.15499 (calculated for C_24_H_31_^35^ClO_9_ + Na, 521.15488).

Briarenol T (**4**): Amorphous powder; ${\left[\alpha \right]}_{D}^{25}$ −30 (*c* 0.1, CHCl_3_); IR (KBr) ν_max_ 3445, 1772, 1740, 1683 cm^−1^; ^13^C (150 MHz, CDCl_3_) and ^1^H (600 MHz, CDCl_3_) NMR data, see Table 1 and Table 2; ESIMS: *m/z* 471 [M + Na]^+^; HRESIMS: *m/z* 471.19879 (calculated for C_24_H_32_O_8_ + Na, 471.19894).

### 3.4. Molecular Mechanics Calculations

The MM2 force field [6] in CHEM3D PRO software from CambridgeSoft Corporation (version 15.0, Cambridge, MA, USA) was used to calculate the molecular models.

### 3.5. In Vitro Anti-inflammatory Assay

The anti-inflammatory assay was employed to evaluate the activities of briaranes **1**‒**4** in reducing the release of iNOS and COX-2 from macrophage cells, as the literature reported [18].

## 4. Conclusions

Four new briaranes, briarenols Q‒T (**1**‒**4**), were identified from a cultured-type octocoral *B. stechei*, which was originally inhabiting and distributed in Taiwanese waters, an area with high biodiversity at the intersection of the Kuroshio current and South China Sea surface current. The structures of compounds **1**‒**4** were elucidated on the basis of spectroscopic analysis and were further confirmed by consideration of biogenetic grounds with modeling compounds that were isolated from the same target cultured octocoral. Briaranes **3** and **4** displayed promising inhibitory effects on the production of iNOS at a concentration of 10 μM. Compound **1** did not exhibit anti-inflammatory activity in this study; more bioactivity screening should be carried out to discover the pharmaceutical potential of the compounds.

## Figures and Tables

**Table 1:** The ^13^C NMR (δ_C_ 150 MHz, CDCl_3_) data for briaranes **1**–**4**.

Position	1	2	3	4
1	44.3, C ^a^	41.1, C	43.9, C	44.1, C
2	72.1, CH	76.0, CH	78.1, CH	79.5, CH
3	41.8, CH_2_	60.2, CH	34.5, CH_2_	31.6, CH_2_
4	94.0, C	57.4, CH	74.2, CH	28.9, CH_2_
5	138.4, C	133.6, C	n. o. ^c^	147.2, C
6	128.0, CH	60.9, CH	62.3, CH	118.1, CH
7	70.1, CH	76.3, CH	77.2, CH	77.7, CH
8	80.8, C	84.2, C	85.0, C	82.2, C
9	77.2, CH	68.4, CH	74.2, CH	71.3, CH
10	40.7, CH	39.2, CH	38.8, CH	38.3, CH
11	131.1, C	44.7, CH	47.3, CH	48.5, CH
12	122.0, CH	201.6, C	202.4, C	202.6, C
13	28.4, CH_2_	124.4, CH	124.5, CH	124.1, CH
14	71.8, CH	152.5, CH	155.1, CH	154.6, CH
15	13.4, CH_3_	14.6, CH_3_	18.1, CH_3_	15.4, CH_3_
16	164.6, C	120.3, CH_2_	122.8, CH_2_	28.4, CH_3_
17	48.0, CH	45.5, CH	45.4, CH	42.6, CH
18	8.7, CH_3_	6.2, CH_3_	7.7, CH_3_	6.8, CH_3_
19	174.9, C	173.7, C	175.6, C	175.5, C
20	24.2, CH_3_	15.6, CH_3_	15.3, CH_3_	15.0, CH_3_
OAc-2	169.2, C ^b^ 21.3, CH_3_	169.6, C 20.9, CH_3_	169.8, C 21.0, CH_3_	168.9, C 21.7, CH_3_
OAc-4			169.1, C 21.0, CH_3_	
OAc-9	169.7, C 21.0, CH_3_	169.3, C 21.8, CH_3_		170.2, C 21.1, CH_3_
OAc-14	173.2, C ^b^ 21.3, CH_3_			
OCH_3_-16	52.4, CH_3_			

^a^ Multiplicity deduced by ^13^C and HSQC spectra; ^b^ data exchangeable; ^c^ n. o. = not obersved.

**Table 2:** The ^1^H NMR (δ_H_ 600 MHz, CDCl_3_) data (*J* in Hz) for briaranes **1**–**4**.

Position	1	2	3	4
2	5.11 d (7.2)	4.75 d (9.0)	4.63 dd (3.6, 3.0)	4.44 dd (6.6, 1.2)
3α	2.46 d (16.8)		1.86 ddd (15.6, 3.6, 3.6)	1.70 m
β	3.40 dd (16.8, 7.2)	3.48 dd (9.0, 4.2)	3.00 ddd (15.6, 12.0, 3.0)	2.75 m
4α			5.81 dd (12.0, 3.6)	2.06 ddd (14.4, 14.4, 4.8)
β		3.67 d (4.2)		2.54 m
6	6.84 d (4.8)	5.39 m	5.29 br s	5.44 br d (10.2)
7	4.44 d (4.8)	5.08 d (3.6)	5.41 d (3.0)	5.23 d (10.2)
9	6.05 s	5.57 d (8.4)	3.90 dd (6.0, 6.0)	5.30 d (4.8)
10	3.10 br s	2.51 dd (8.4, 4.2)	2.67 br s	2.69 dd (4.8, 4.2)
11		2.87 qd (7.2, 4.2)	2.45 m	2.50 qd (7.2, 4.2)
12	5.56 br s			
13α/β	2.02 m; 2.38 br d (18.0)	5.88 dd (10.2, 0.6)	5.89 d (10.2)	5.85 d (10.2)
14	5.18 d (4.2)	6.37 d (10.2)	6.47 d (10.2)	6.39 d (10.2)
15	1.04 s	1.28 s	1.46 s	1.23 s
16a/b		5.79 d (3.0); 6.06 d (3.0)	5.72 s; 5.89 s	1.99 d (1.2)
17	2.79 q (7.2)	2.50 q (7.2)	3.05 q (7.8)	2.44 q (7.2)
18	1.46 d (7.2)	1.25 d (7.2)	1.19 d (7.8)	1.21 d (7.2)
20	1.96 br s	1.30 d (7.2)	1.25 d (7.2)	1.32 d (7.2)
OH-4	6.11 s			
OH-8		3.52 s	3.42 s	n. o. ^b^
OAc-2	2.04 s ^a^	2.23 s	2.09 s ^a^	2.24 s
OAc-4			2.17 s ^a^	
OAc-9	2.03 s	2.27 s		2.13 s
OAc-14	2.11 s ^a^			
OCH_3_-16	3.81 s			

^a^ Data exchangeable; ^b^ n. o. = not observed.

**Table 3:** Western blotting showed that briaranes **3** and **4** suppressed the expression of iNOS. Data were normalized to the cells treated with LPS only, while cells treated with dexamethasone (Dex) (10 μM) were used as a positive control.

Compound	iNOS	COX-2
	Expression (% of LPS) at 10 μM	
Control	1.28 ± 0.29	0.76 ± 0.13
LPS	100.00 ± 1.87	100.00 ± 3.26
**1**	98.27 ± 5.13	94.00 ± 3.47
**2**	84.53 ± 4.66 *	112.96 ± 4.54
**3**	78.50 ± 3.45 *	97.66 ± 4.60
**4**	79.95 ± 2.94 *	104.66 ± 7.86
Dexamethasone	24.56 ± 1.85 *	6.56 ± 1.18 *

Data are presented as the mean ± SEM (*n* = 4); * significantly different from cells treated with LPS (*p* < 0.05).

## Supplementary Materials

Supplementary Materials are available online at https://www.mdpi.com/1660-3397/18/8/383/s1. ESIMS, HRESIMS, IR, 1D (^1^H and ^13^C) and 2D (HSQC, HMBC, COSY, and NOESY) NMR spectra of briarenols Q–T (**1**–**4**).
