# Supplementary material for: Briarenols Q–T: Briaranes from A Cultured Octocoral Briareum stechei (Kükenthal, 1908)
Source: Mar Drugs. 2020 Jul 24;18(8):383. doi: 10.3390/md18080383 (PMC7460508; doi:10.3390/md18080383)
Supplement: Supplementary file 1 [file marinedrugs-18-00383-s001.pdf]

S1. ESIMS spectrum of compound 1..2
S2. HRESIMS spectrum of compound **1**..3
S3. IR spectrum of compound 1..3
S4. ^{1}H NMR spectrum (600 MHz) of compound 1 in $CDCl_3$...4
S5. ^{13}C NMR spectrum (150 MHz) of compound 1 in $CDCl_3$...4
S6. HSQC spectrum of compound 1 in $CDCl_3$...5
S7. HMBC spectrum of compound 1 in $CDCl_3$..5
S8. ^{1}H-^{1}H COSY spectrum of compound 1 in $CDCl_3$..6
S9. NOESY spectrum of compound 1 in $CDCl_3$..6
S10. ESIMS spectrum of compound **2**...7
S11. HRESIMS spectrum of compound 2..8
S12. IR spectrum of compound 2 8
S13. ^{1}H NMR spectrum (600 MHz) of compound 2 in $CDCl_3$...9
S14. ^{13}C NMR spectrum (150 MHz) of compound 2 in $CDCl_3$...9
S15. HSQC spectrum of compound 2 in $CDCl_3$...10
S16. HMBC spectrum of compound 2 in $CDCl_3$...10
S17. ^{1}H-^{1}H COSY spectrum of compound 2 in $CDCl_3$..11
S18. NOESY spectrum of compound 2 in $CDCl_3$...11
S19. ESIMS spectrum of compound 3..12
S20. HRESIMS spectrum of compound **3**...13
S21. IR spectrum of compound 3...13
S22. ^{1}H NMR spectrum (600 MHz) of compound 3 in $CDCl_3$.......................................14
S23. ^{13}C NMR spectrum (150 MHz) of compound 3 in $CDCl_3$.......................................14
S24. HSQC spectrum of compound 3 in $CDCl_3$...15
S25. HMBC spectrum of compound 3 in $CDCl_3$...15
S26. ^{1}H-^{1}H COSY spectrum of compound 3 in $CDCl_3$..16
S27. NOESY spectrum of compound 3 in $CDCl_3$...16
S28. ESIMS spectrum of compound **4**...17
S29. HRESIMS spectrum of compound 4 18
S30. IR spectrum of compound 4 18
S31. ^{1}H NMR spectrum (600 MHz) of compound 4 in $CDCl_3$.......................................19
S32. ^{13}C NMR spectrum (150 MHz) of compound 4 in $CDCl_3$.......................................19
S33. HSQC spectrum of compound 4 in $CDCl_3$...20
S34. HMBC spectrum of compound 4 in $CDCl_3$...20
S35. ^{1}H-^{1}H COSY spectrum of compound 4 in $CDCl_3$..21
S36. NOESY spectrum of compound 4 in $CDCl_3$...21
S37. Western blotting of 1–4..22

FT-MS

Analysis Info

Analysis Name D:\Data\a2\bp3265233_000002.d

5/13/2020 4:32:58 PM

Method broadband first signal

Sample Name bp3-2-6-5-2-3-3

Instrument: FT-MS solariX

Comment ESI Positive

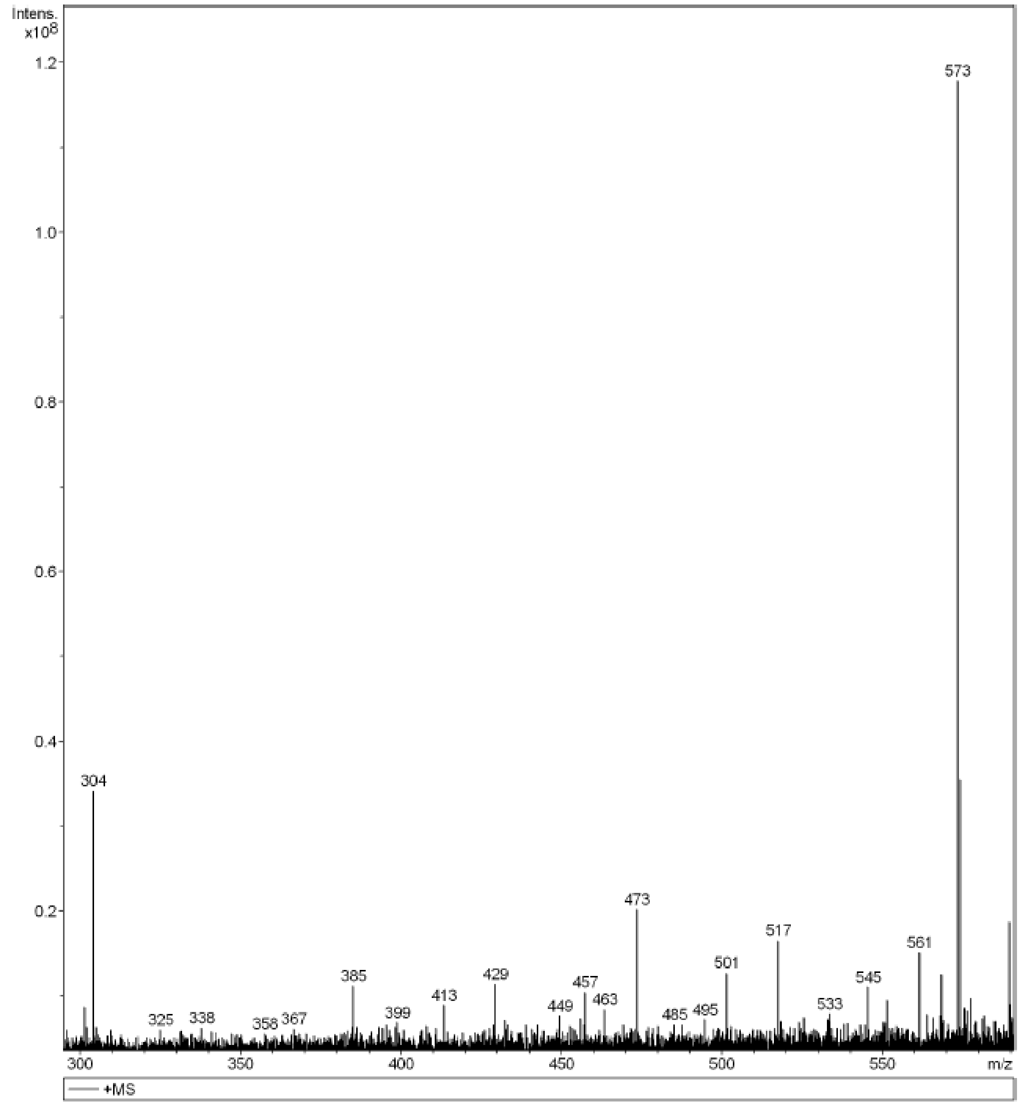

Bruker Compass DataAnalysis 4.0 printed: 5/13/2020 4:32:58 PM Page 1 of 1

S1. ESIMS spectrum of compound **1**

Mass Spectrum SmartFormula Report

Analysis Info

Analysis Name	D:\Data\a2\bp3265233_000001.d	5/13/2020 4:25:57 PM
Method	broadband first signal	Operator: YU HSIAO-CHING
Sample Name	bp3-2-6-5-2-3-3	Instrument: BRUKER FT-MS solariX
Comment	ESI Positive	

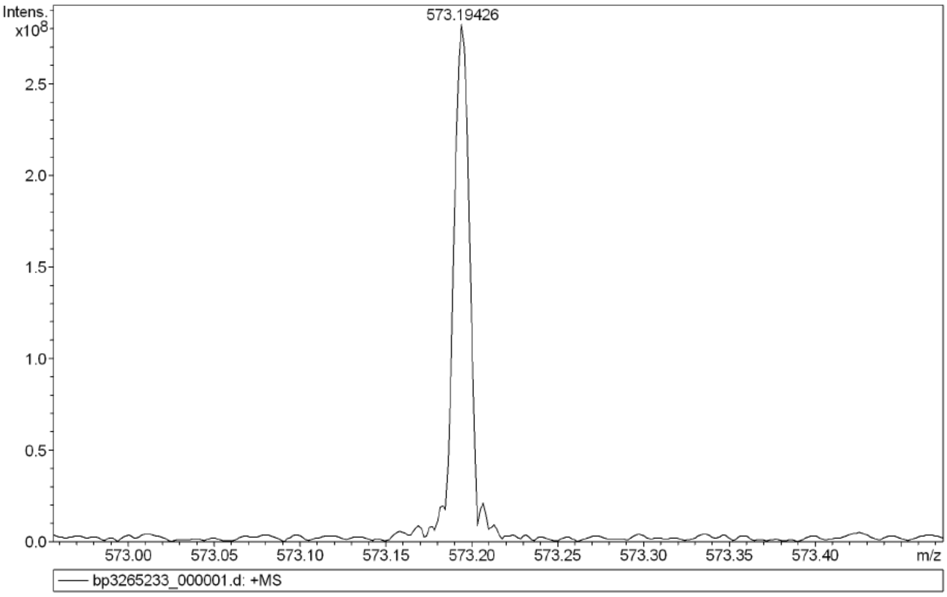

Meas. m/z	#	Formula	Score	m/z	err [mDa]	err [ppm]	mSigma	rdb	e⁻ Conf	N-Rule
573.19426	1	C 27 H 34 Na O 12	100.00	573.19425	-0.01	-0.02	7.4	10.5	even	ok

S2. HRESIMS spectrum of compound **1**

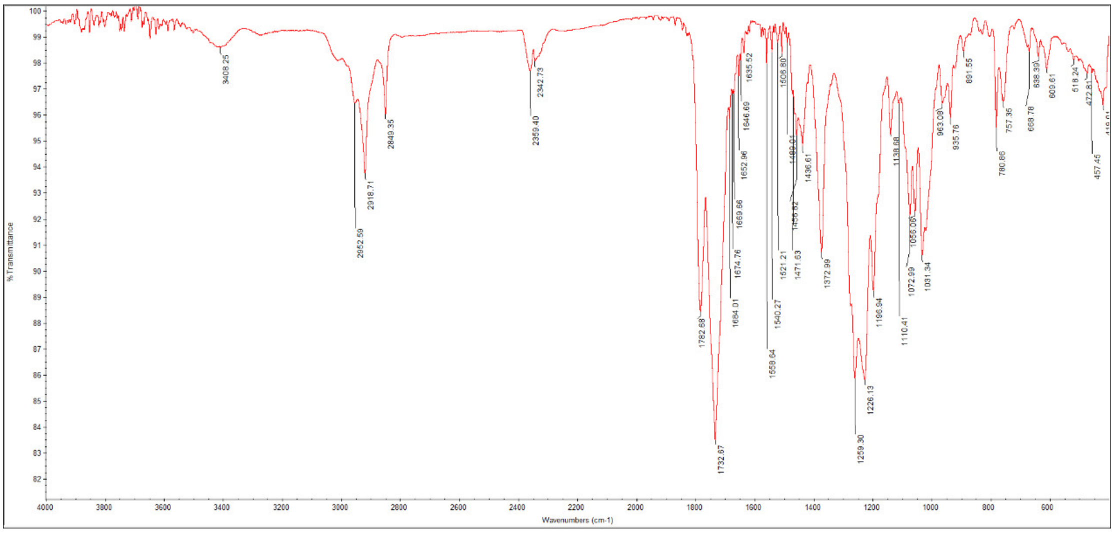

S3. IR spectrum of compound **1**

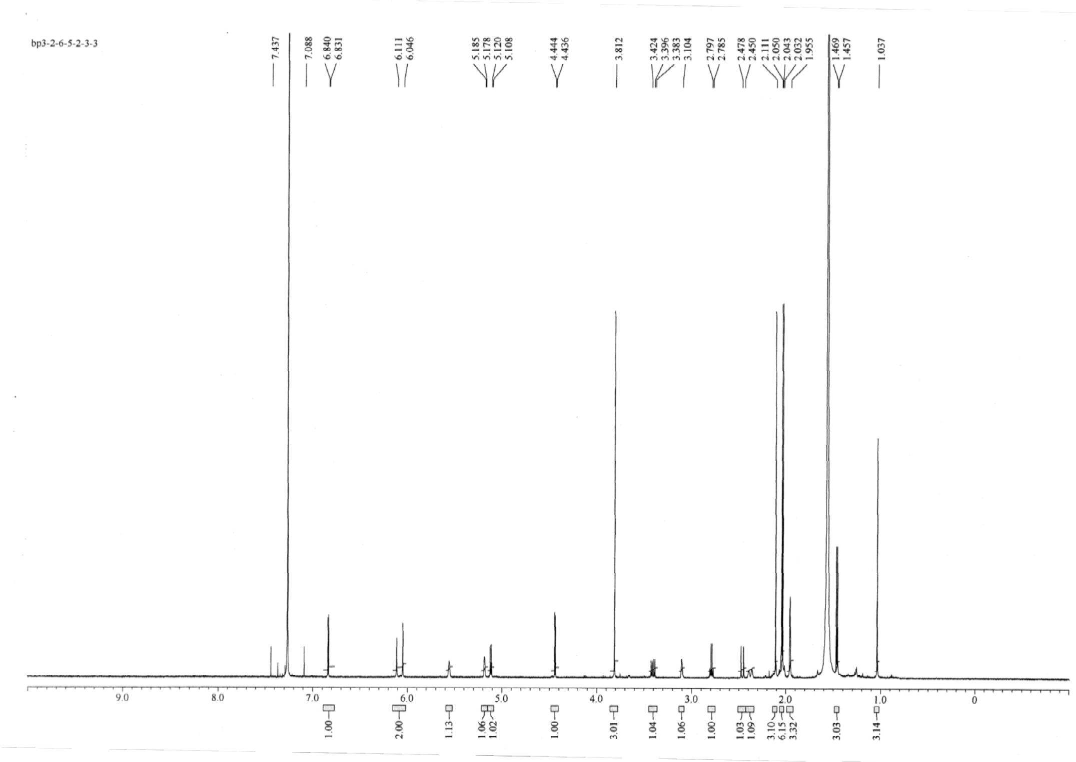

S4. ^{1}H NMR spectrum (600 MHz) of compound **1** in $CDCl_3$

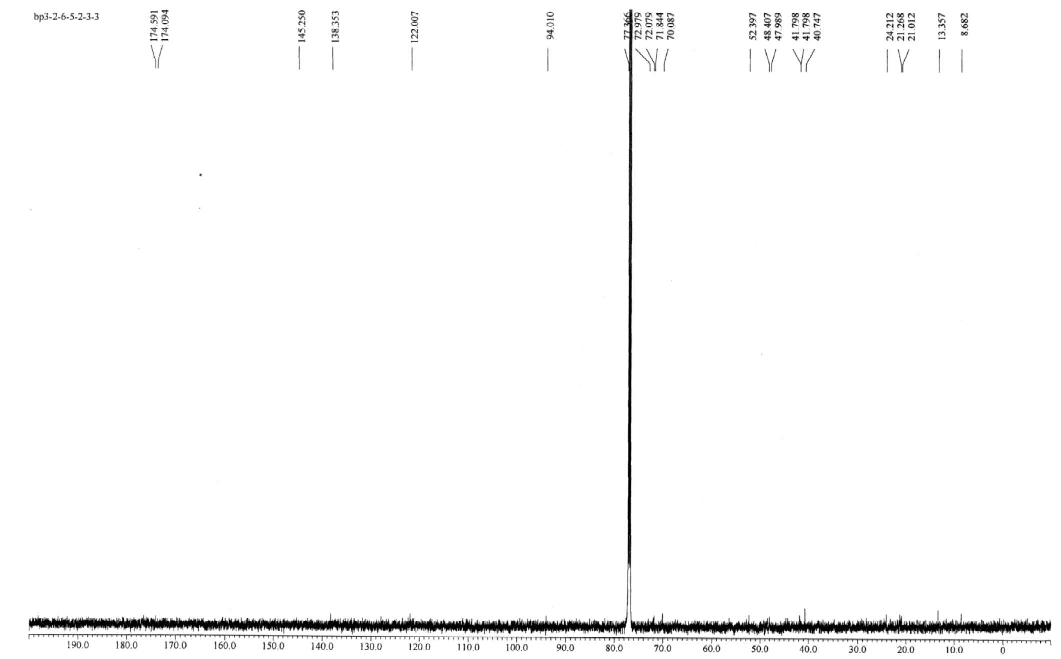

S5. ^{13}C NMR spectrum (150 MHz) of compound **1** in $CDCl_3$

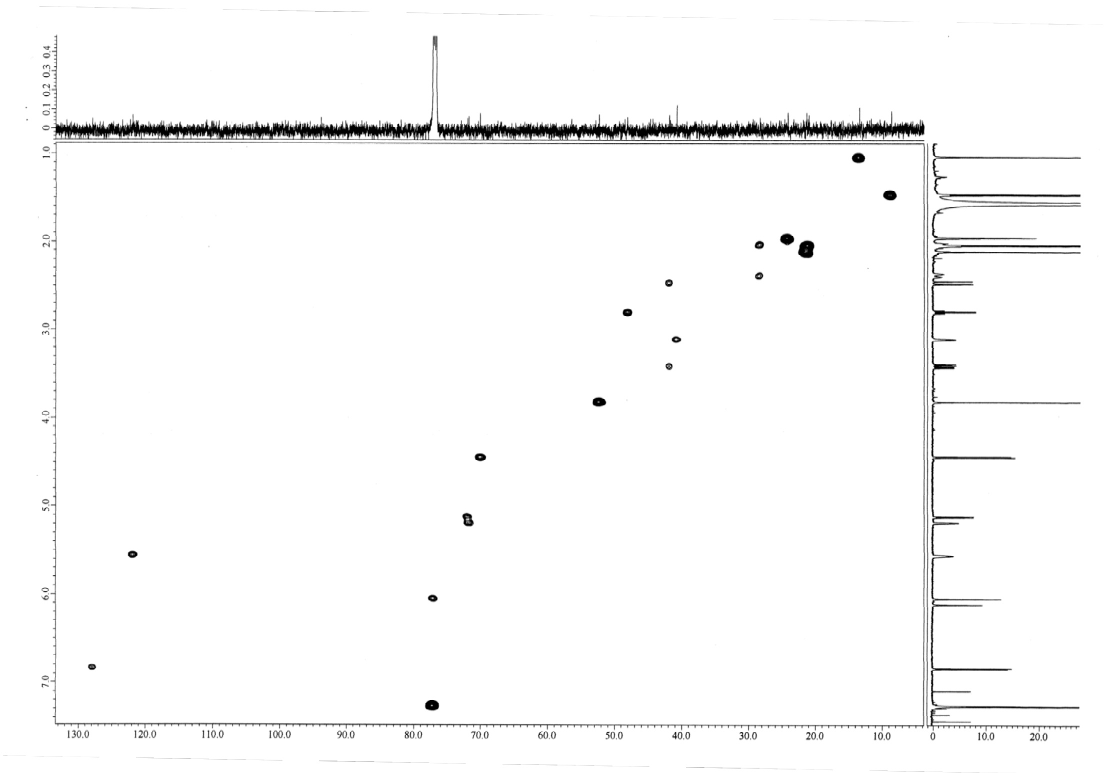

S6. HSQC spectrum of compound **1** in $CDCl_3$

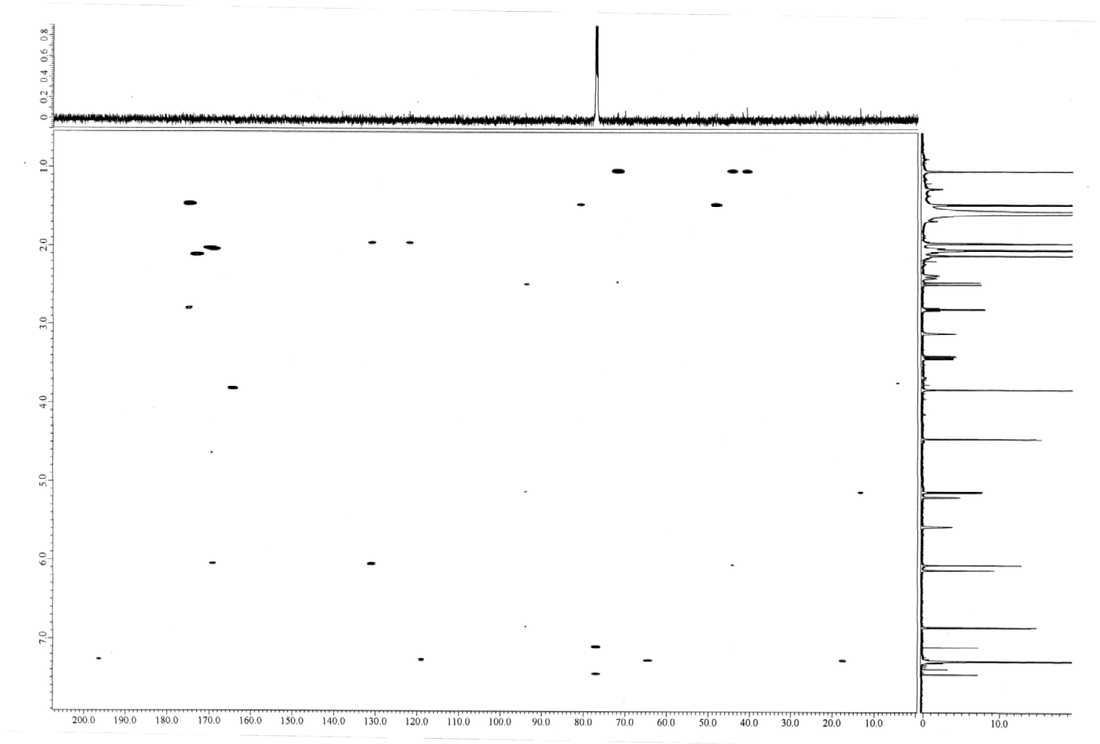

S7. HMBC spectrum of compound **1** in $CDCl_3$

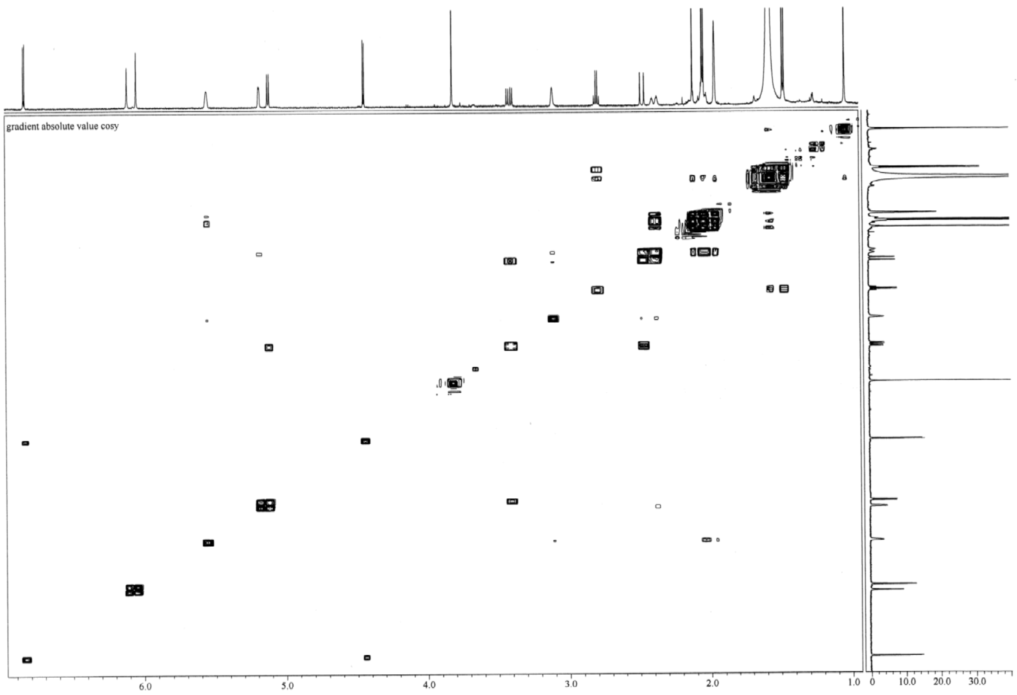

S8. ^{1}H-^{1}H COSY spectrum of compound **1** in $CDCl_3$

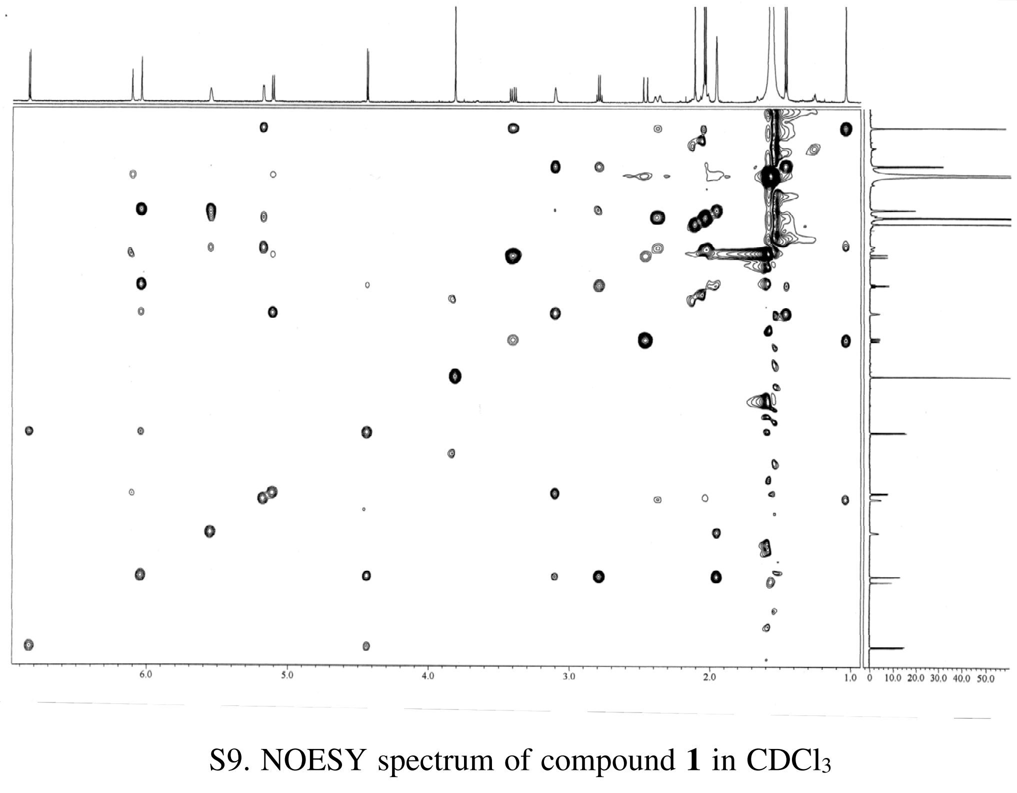

S9. NOESY spectrum of compound **1** in $CDCl_3$

FT-MS

Analysis Info

Analysis Name	D:\Data\a2\BP3265231_000004.d	5/15/2020 12:25:33 PM
Method	broadband first signal	
Sample Name	bp3-2-6-5-2-3-1	Instrument: FT-MS solariX
Comment	ESI Positive	

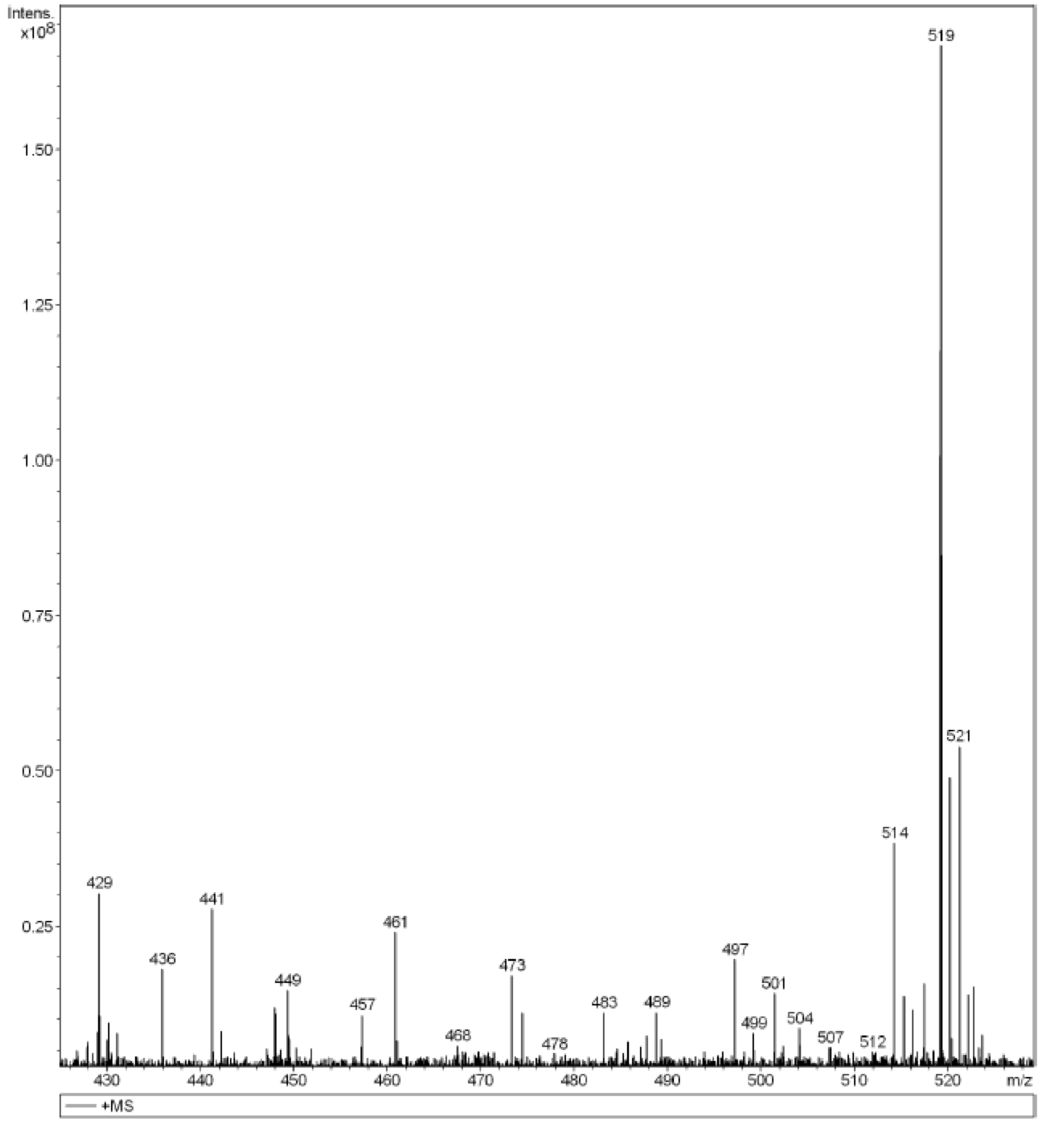

Bruker Compass DataAnalysis 4.0 printed: 5/15/2020 12:25:33 PM Page 1 of 1

S10. ESIMS spectrum of compound **2**

Mass Spectrum SmartFormula Report

Analysis Info

Analysis Name	D:\Data\a2\BP3265231_000003.d	5/15/2020 12:24:21 PM
Method	broadband first signal	Operator: YU HSIAO-CHING
Sample Name	bp3-2-6-5-2-3-1	Instrument: BRUKER FT-MS solariX
Comment	ESI Positive	

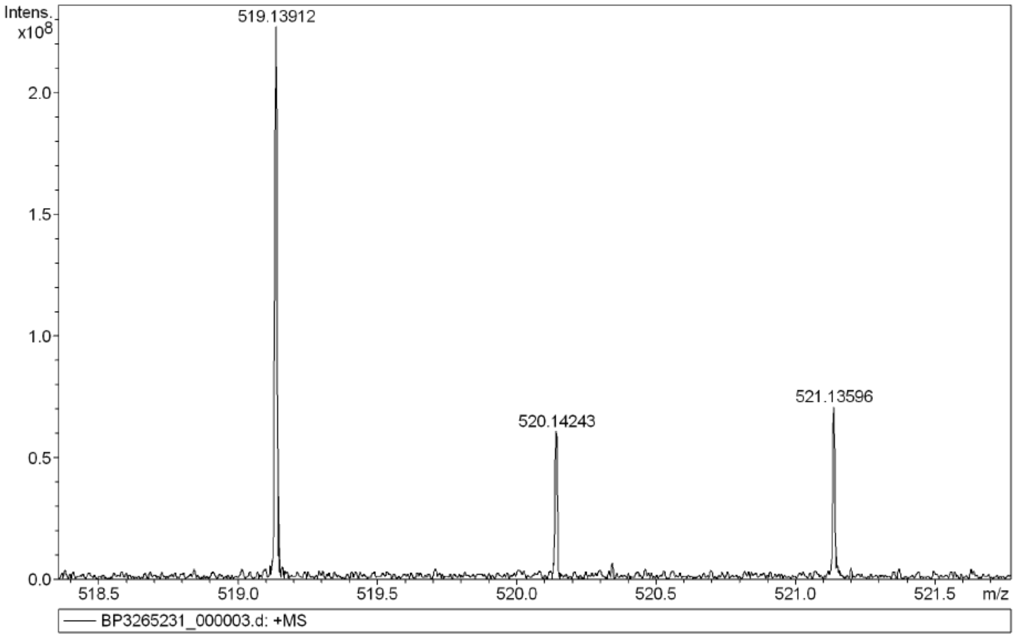

Meas. m/z	#	Formula	Score	m/z	err [mDa]	err [ppm]	mSigma	rdb	e⁻ Conf	N-Rule
519.13912	1	C 24 H 29 Cl Na O 9	100.00	519.13923	0.11	0.22	27.4	9.5	even	ok

S11. HRESIMS spectrum of compound **2**

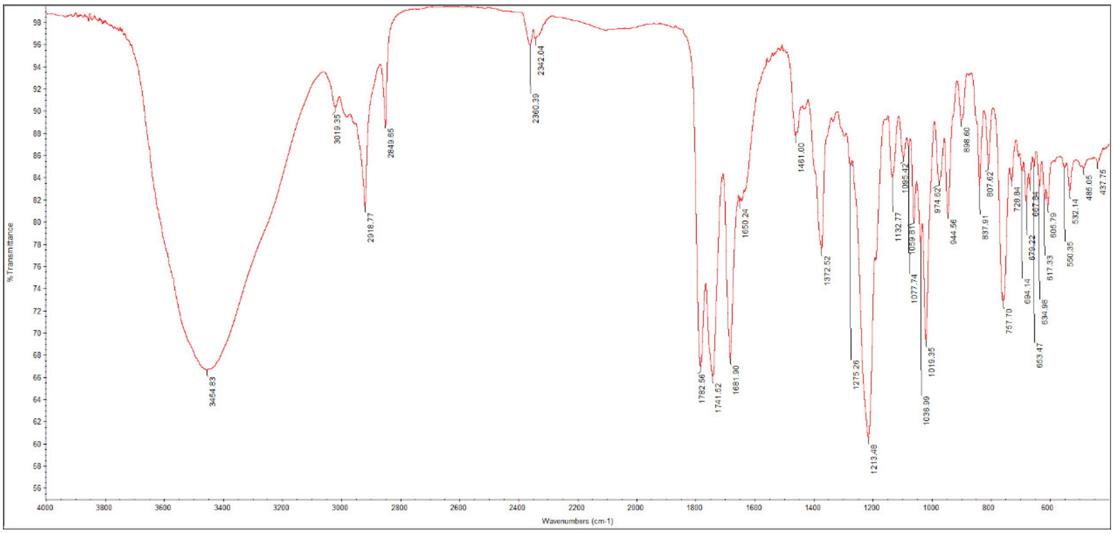

S12. IR spectrum of compound **2**

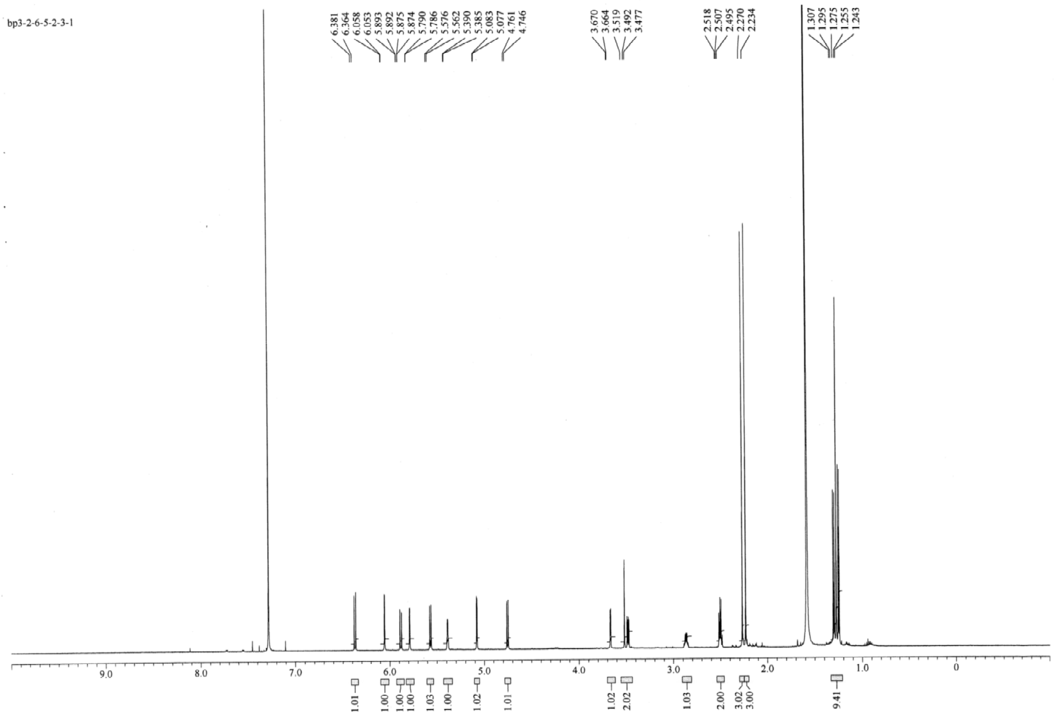

S13. ^{1}H NMR spectrum (600 MHz) of compound **2** in $CDCl_3$

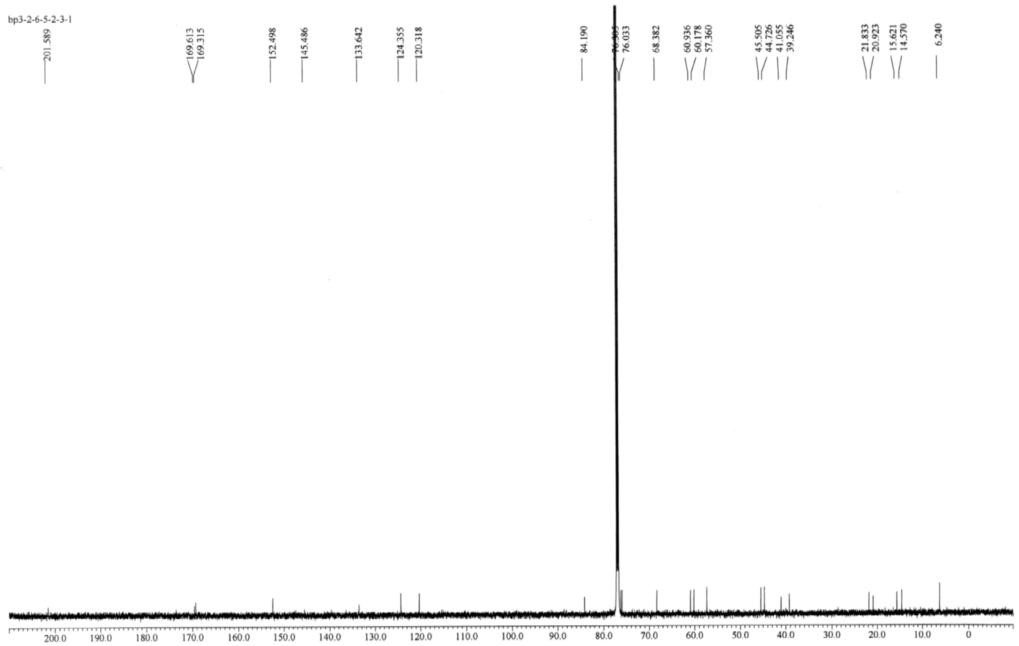

S14. ^{13}C NMR spectrum (150 MHz) of compound **2** in $CDCl_3$

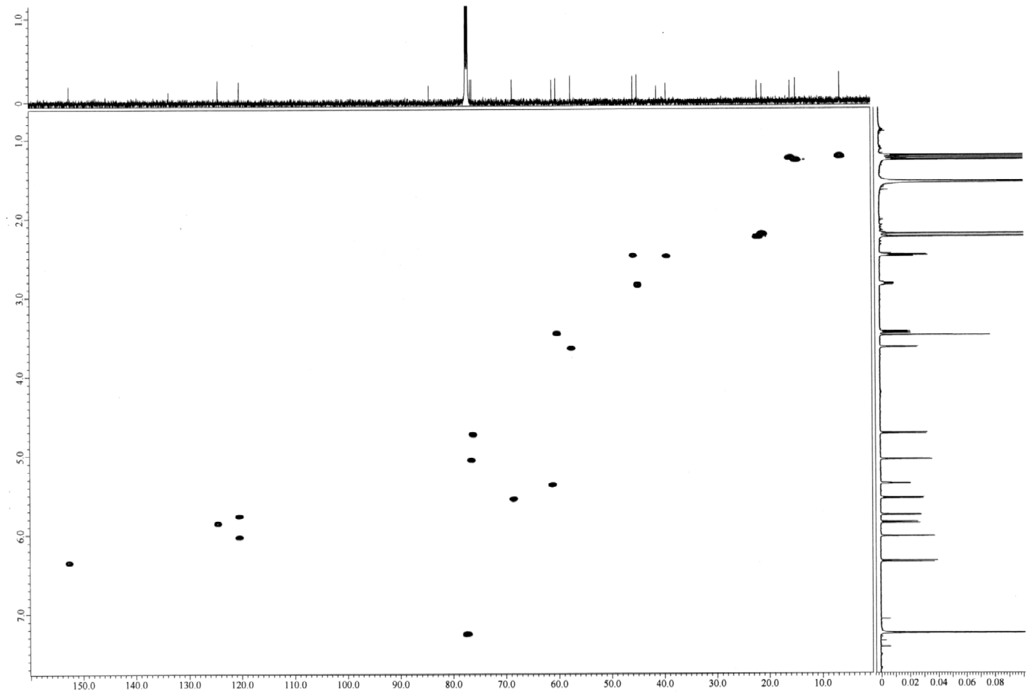

S15. HSQC spectrum of compound **2** in $CDCl_3$

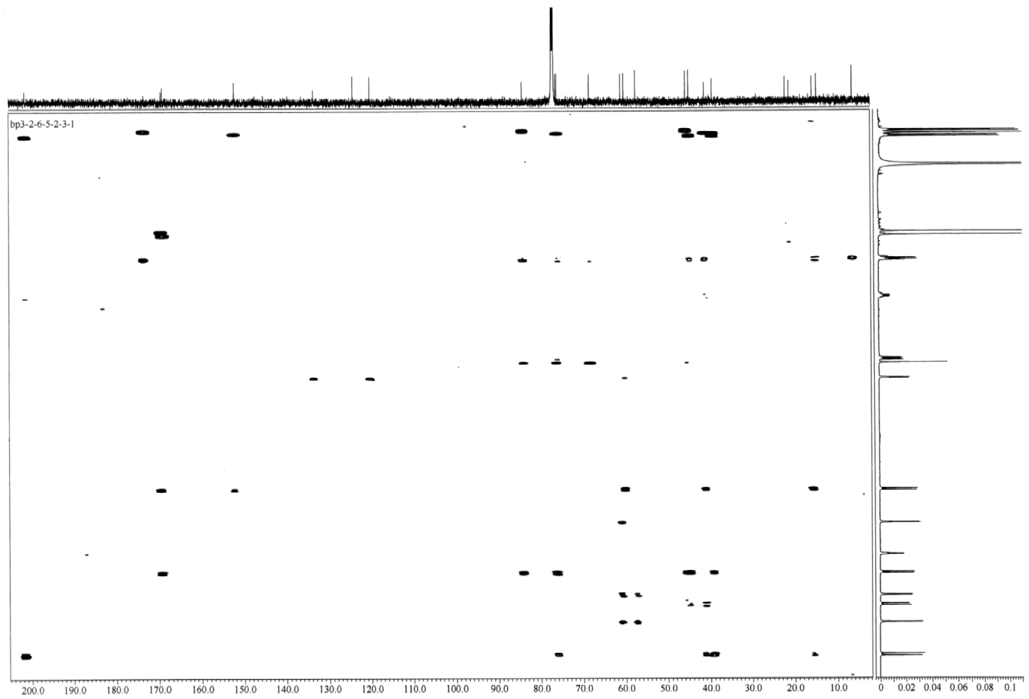

S16. HMBC spectrum of compound **2** in $CDCl_3$

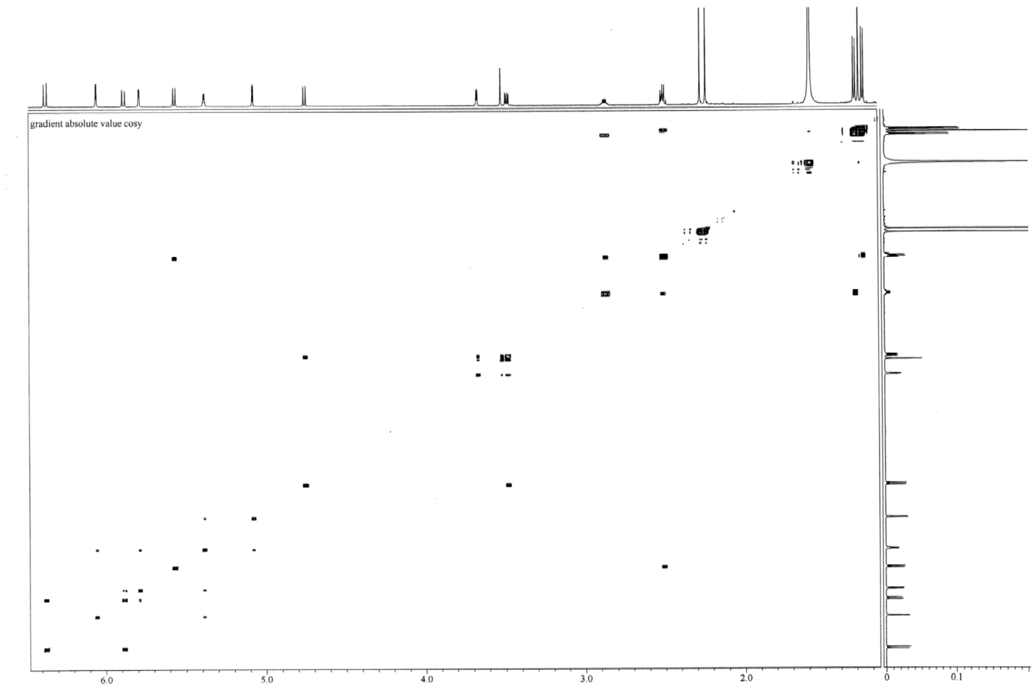

S17. ^{1}H-^{1}H COSY spectrum of compound **2** in $CDCl_3$

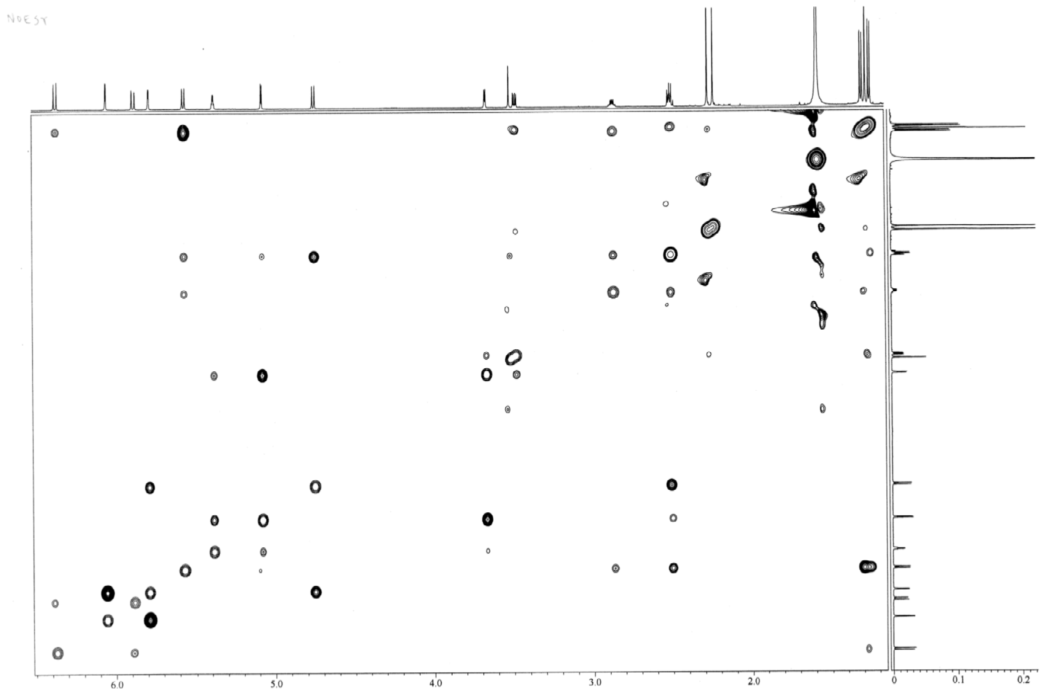

S18. NOESY spectrum of compound **2** in $CDCl_3$

FT-MS

Analysis Info

Analysis Name	D:\Data\a2\bp32673211_000002.d	5/25/2020 2:22:12 PM	
Method	broadband first signal		
Sample Name	bp3-2-6-7-3-2-1-1	Instrument: FT-MS	solariX
Comment	ESI Positive		

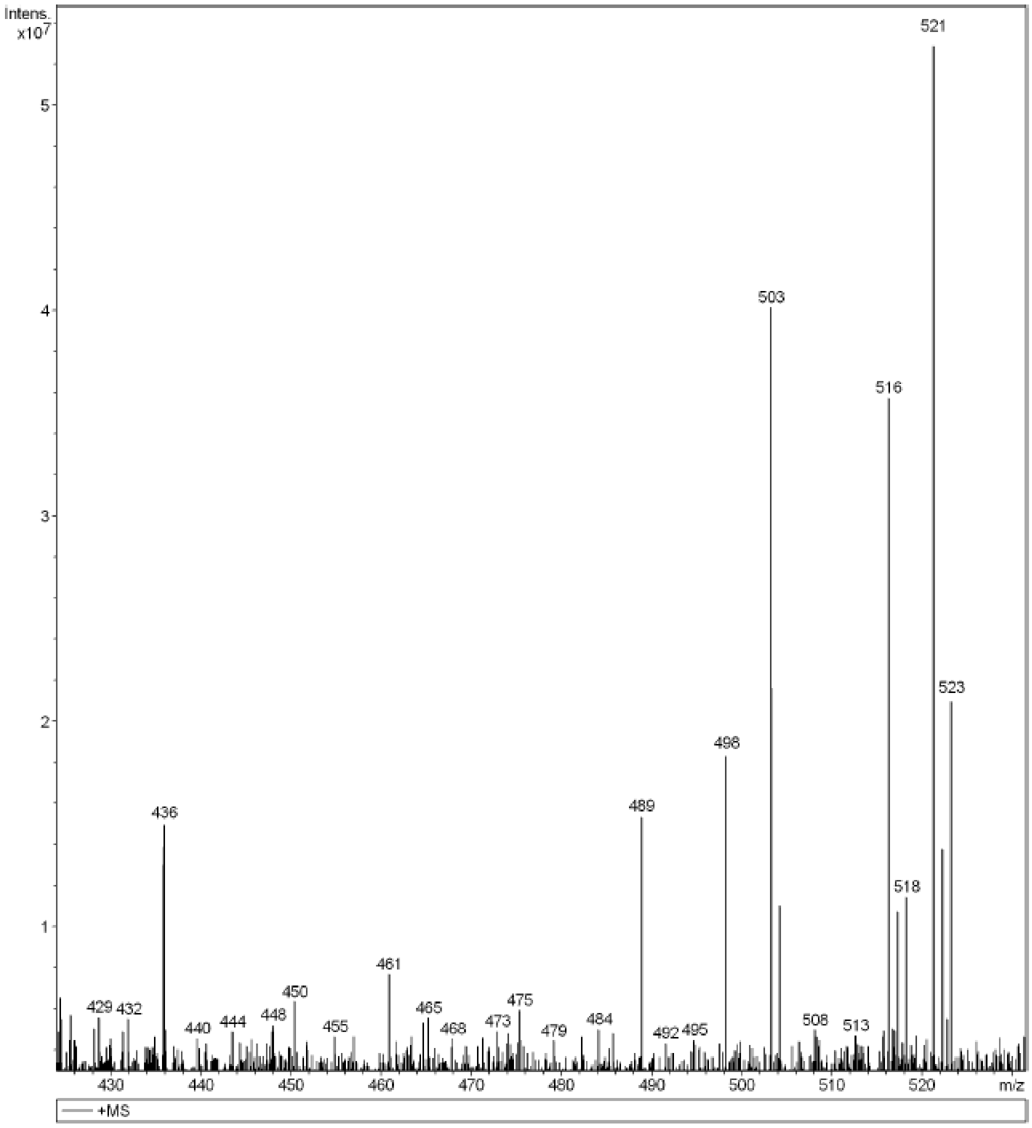

Bruker Compass DataAnalysis 4.0 printed: 5/25/2020 2:22:12 PM Page 1 of 1

S19. ESIMS spectrum of compound **3**

Mass Spectrum SmartFormula Report

Analysis Info

Analysis Name	D:\Data\a2\bp32673211_000002.d	5/25/2020 2:23:53 PM
Method	broadband first signal	Operator: YU HSIAO-CHING
Sample Name	bp3-2-6-7-3-2-1-1	Instrument: BRUKER FT-MS solariX
Comment	ESI Positive	

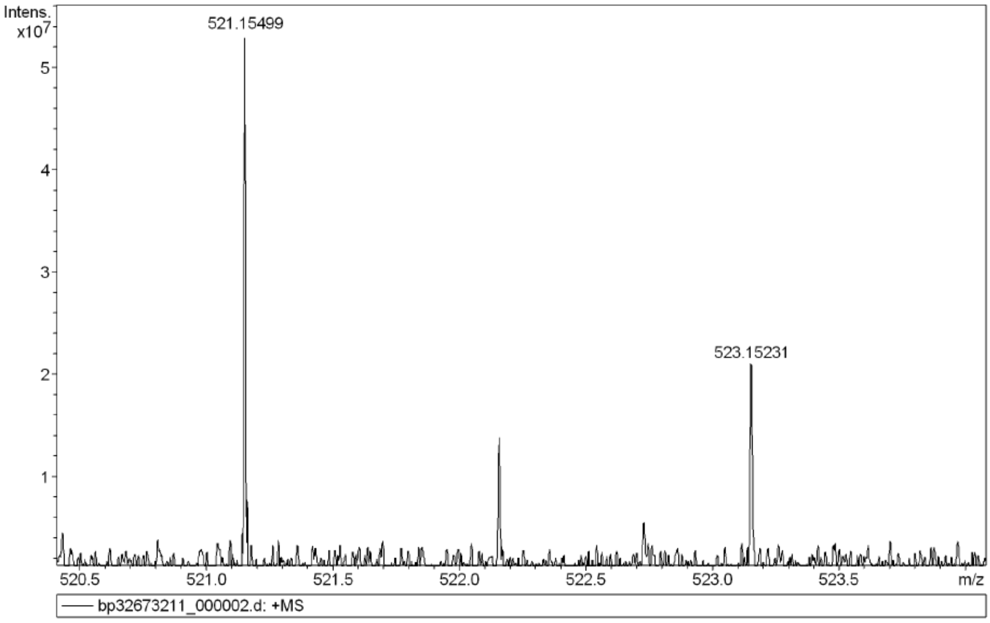

Meas. m/z	#	Formula	Score	m/z	err [mDa]	err [ppm]	mSigma	rdb	e⁻ Conf	N-Rule
521.15499	1	C 24 H 31 Cl Na O 9	100.00	521.15488	-0.11	-0.21	42.2	8.5	even	ok

S20. HRESIMS spectrum of compound **3**

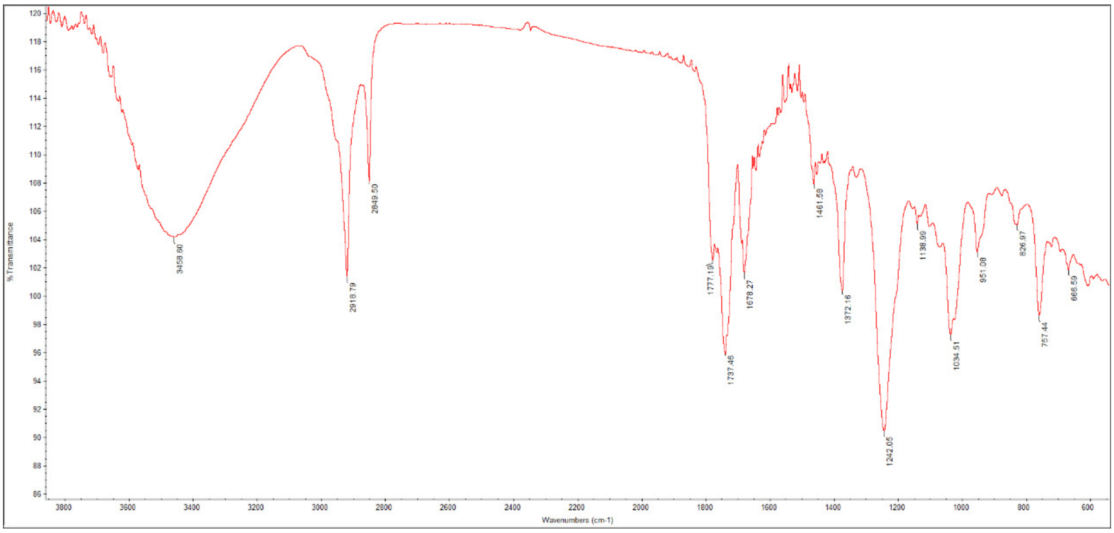

S21. IR spectrum of compound **3**

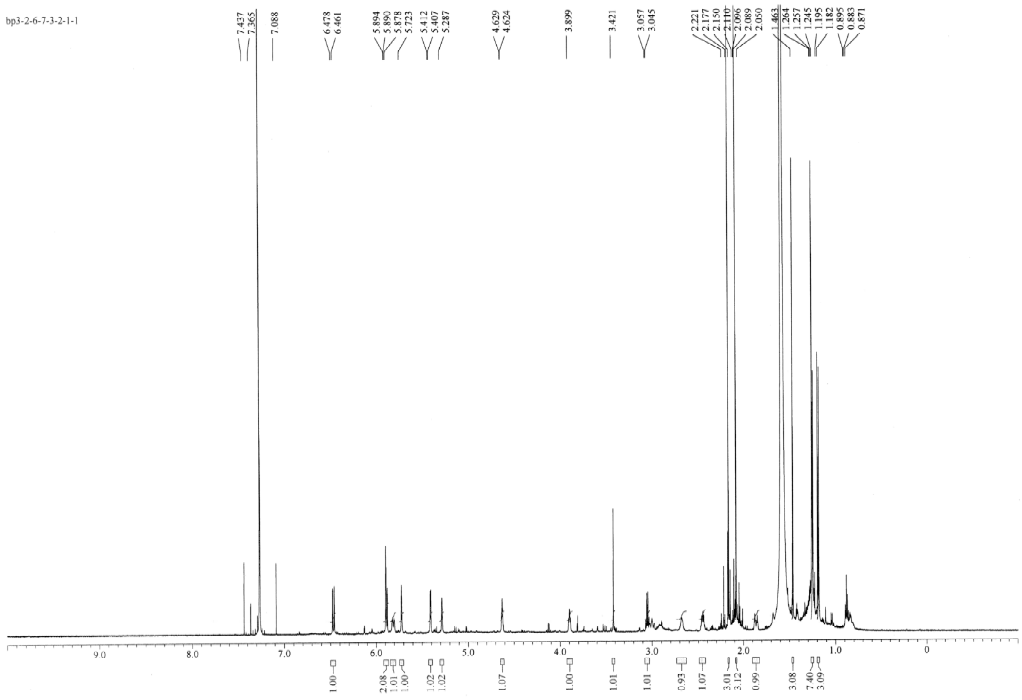

S22. ^{1}H NMR spectrum (600 MHz) of compound **3** in $CDCl_3$

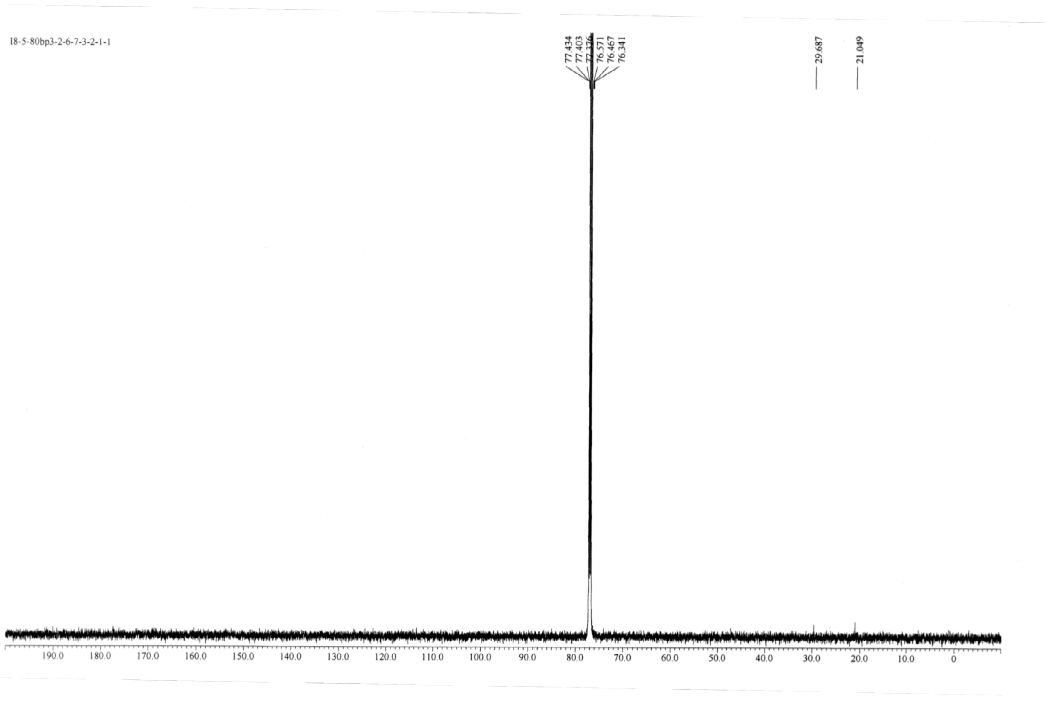

S23. ^{13}C NMR spectrum (150 MHz) of compound **3** in $CDCl_3$

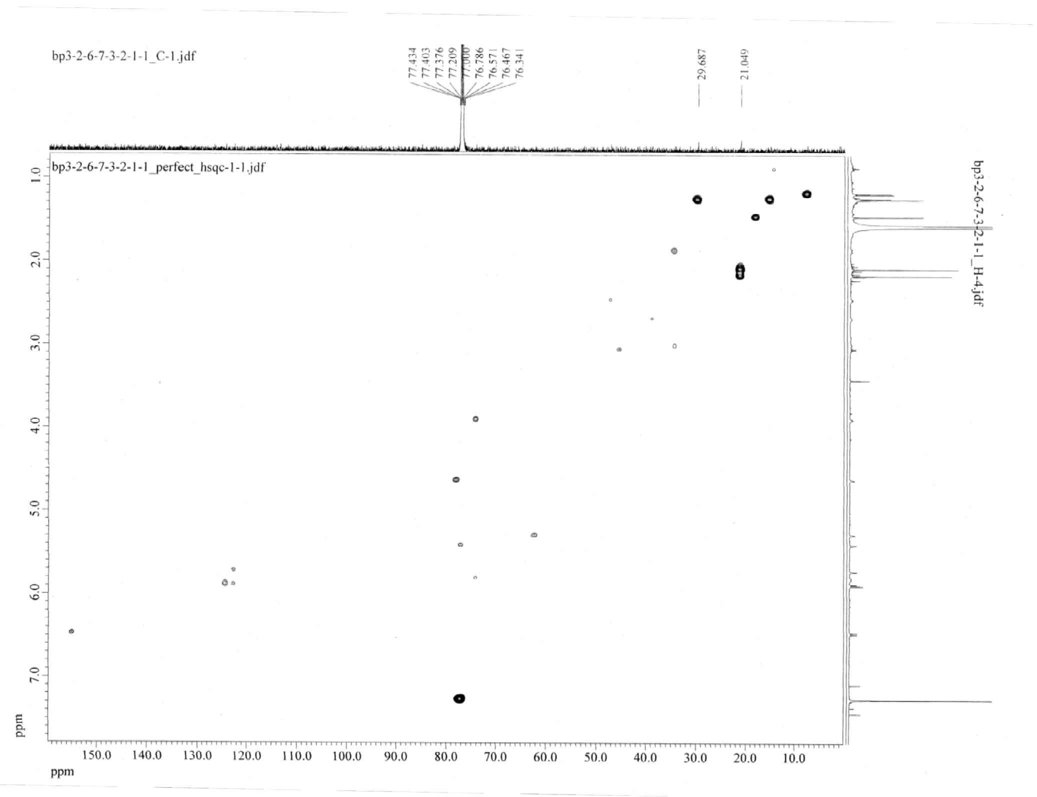

S24. HSQC spectrum of compound **3** in $CDCl_3$

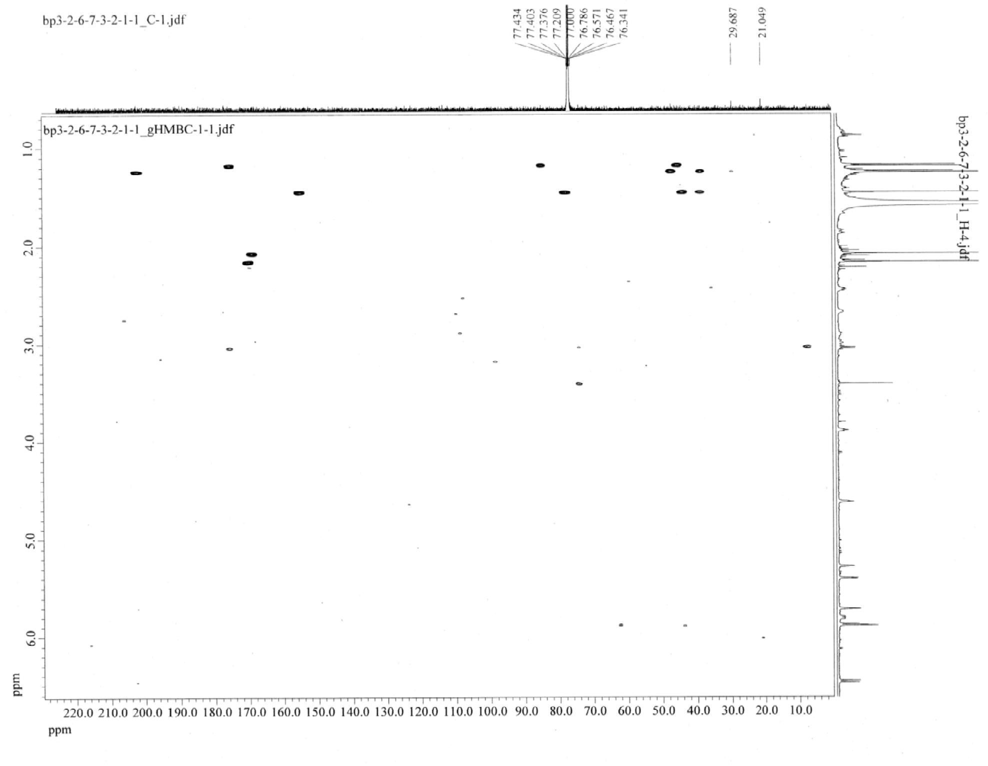

S25. HMBC spectrum of compound **3** in $CDCl_3$

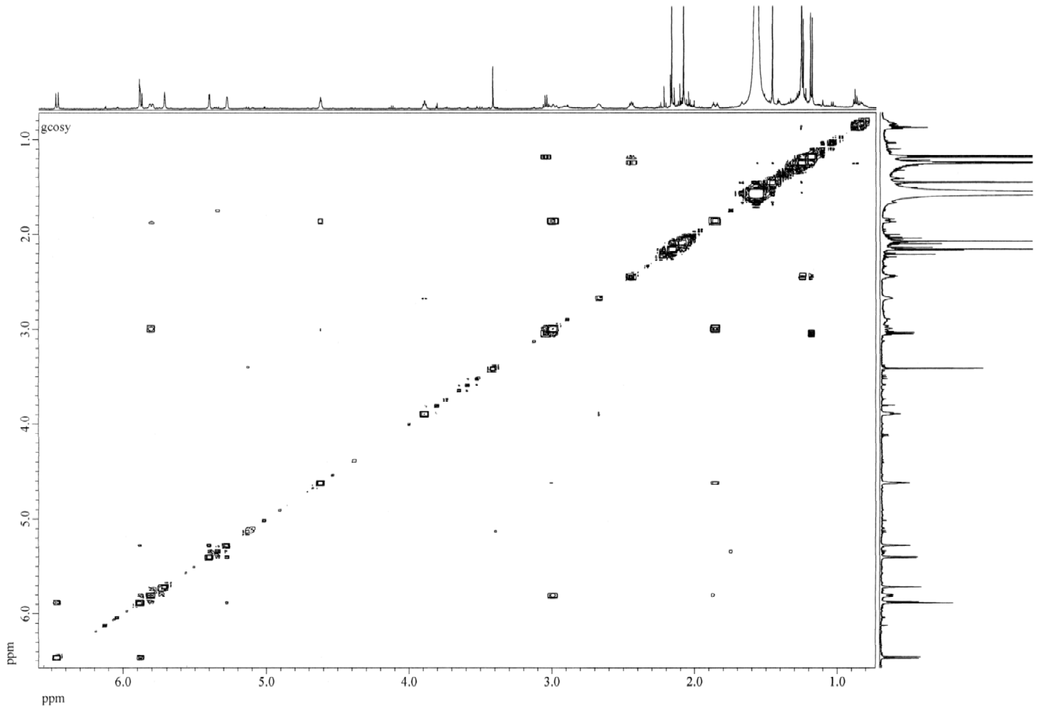

S26. ^{1}H-^{1}H COSY spectrum of compound **3** in $CDCl_3$

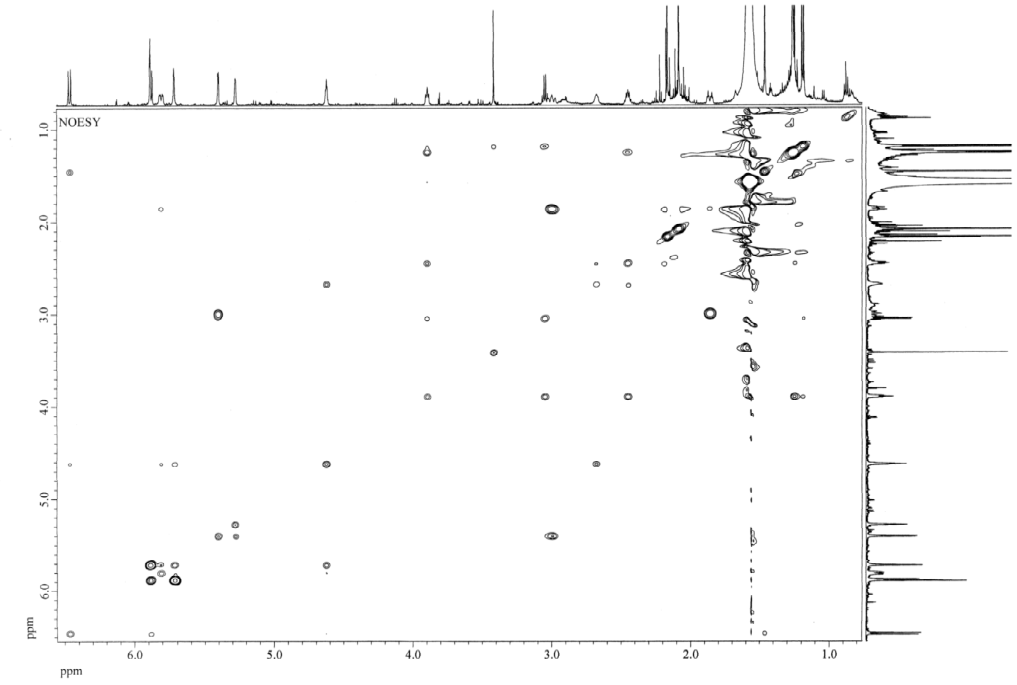

S27. NOESY spectrum of compound **3** in $CDCl_3$

FT-MS

Analysis Info

Analysis Name D:\Data\a2\bp3265232_000001.d
Method broadband first signal
Sample Name bp3-2-6-5-2-3-2
Comment ESI Positive

5/13/2020 4:45:37 PM
Instrument: FT-MS solariX

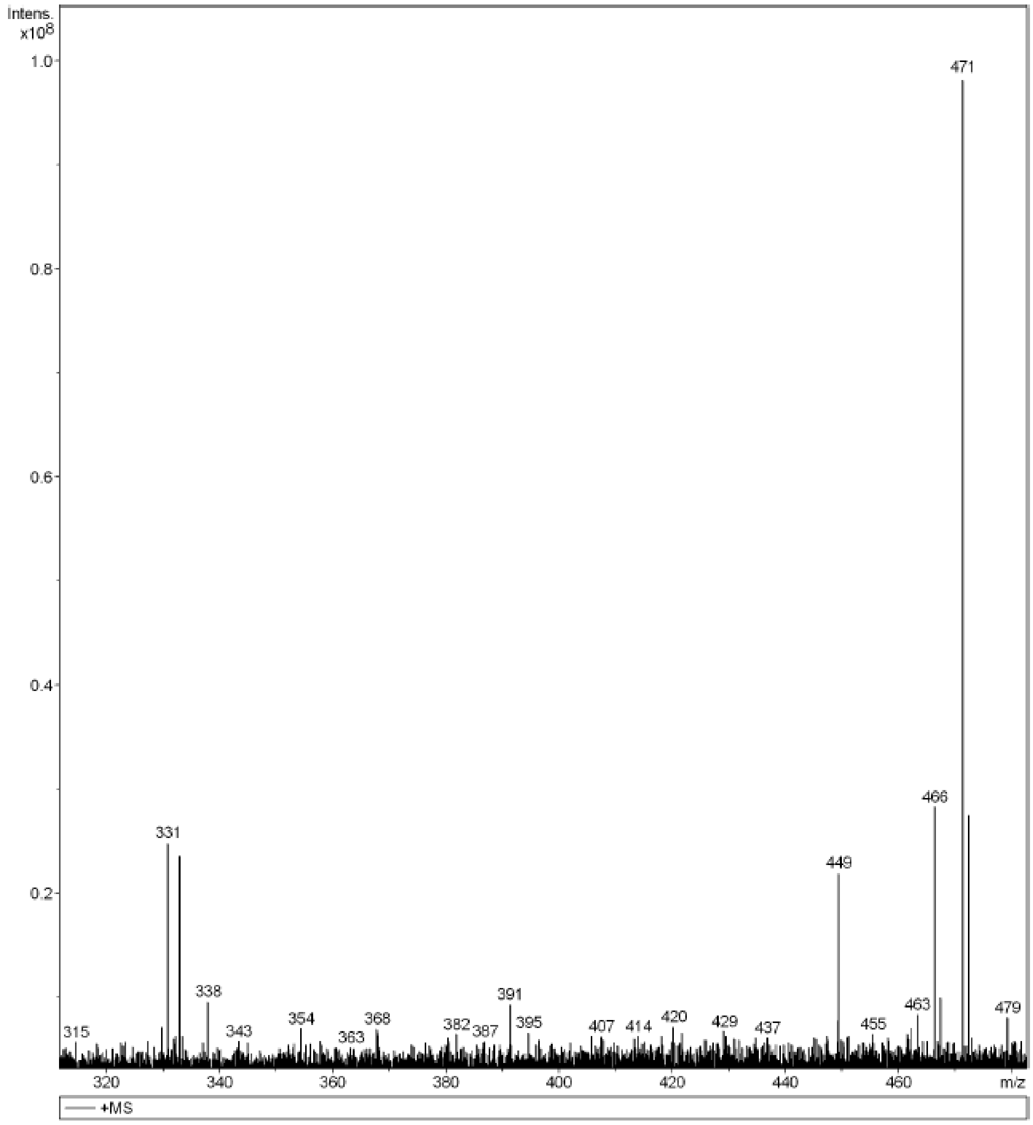

Bruker Compass DataAnalysis 4.0 printed: 5/13/2020 4:45:37 PM Page 1 of 1

S28. ESIMS spectrum of compound **4**

Mass Spectrum SmartFormula Report

Analysis Info

Analysis Name	D:\Data\a2\bp3265232_000002.d	5/13/2020 4:42:47 PM
Method	broadband first signal	Operator: YU HSIAO-CHING
Sample Name	bp3-2-6-5-2-3-2	Instrument: BRUKER FT-MS solariX
Comment	ESI Positive	

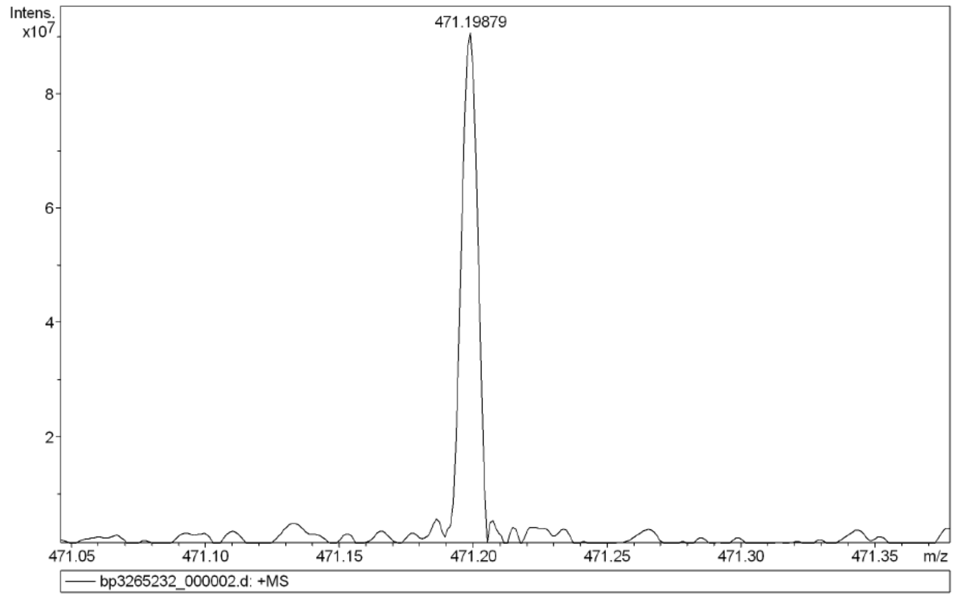

Meas. m/z	#	Formula	Score	m/z	err [mDa]	err [ppm]	mSigma	rdb	e⁻ Conf	N-Rule
471.19879	1	C 24 H 32 Na O 8	100.00	471.19894	0.15	0.31	25.0	8.5	even	ok

S29. HRESIMS spectrum of compound **4**

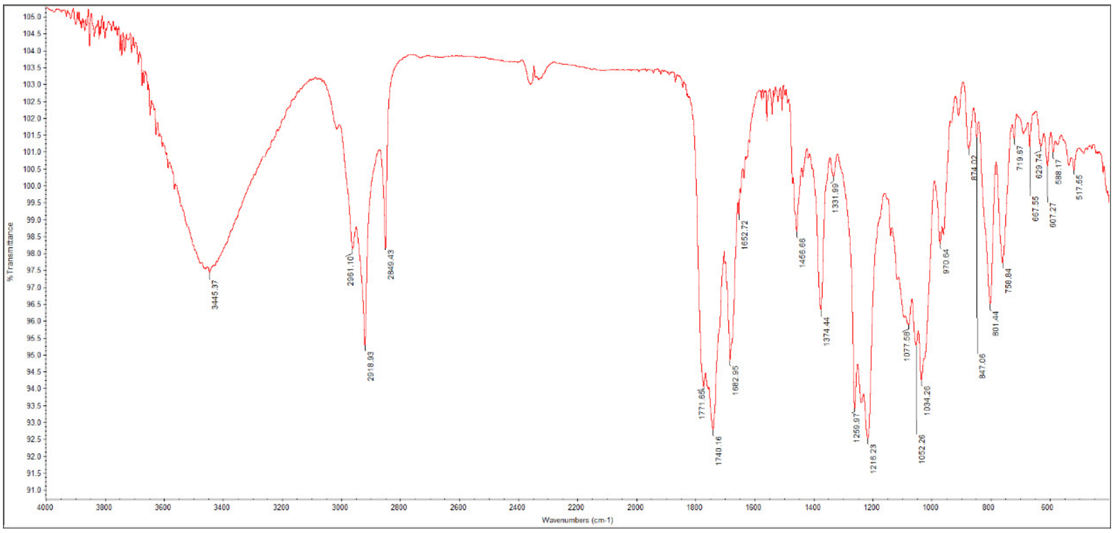

S30. IR spectrum of compound **4**

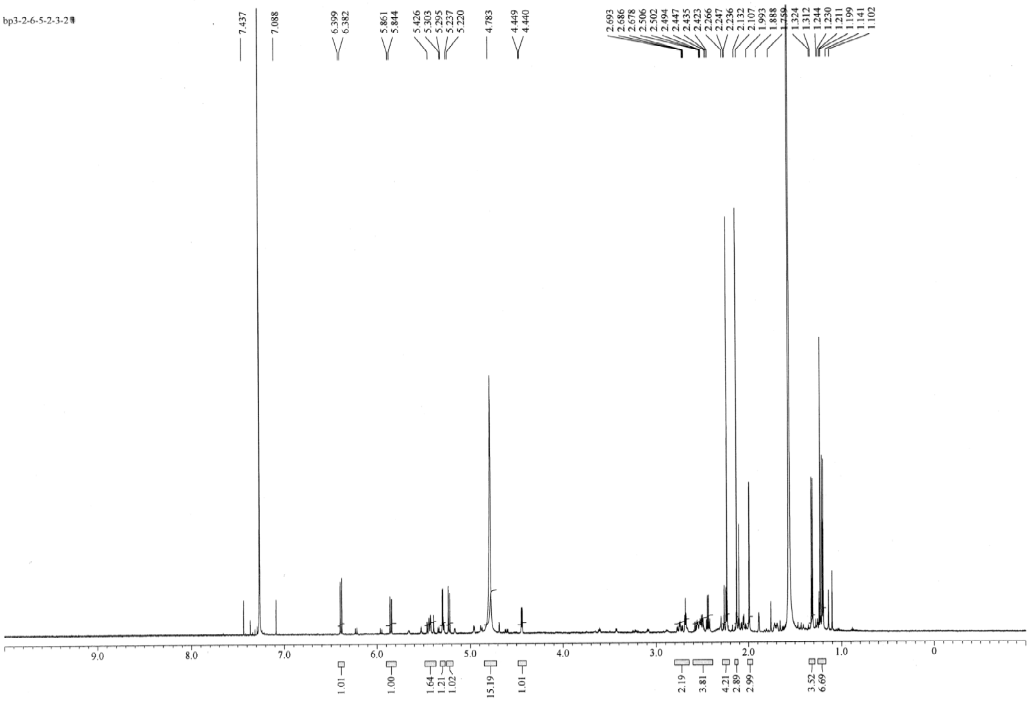

S31. ^{1}H NMR spectrum (600 MHz) of compound **4** in $CDCl_3$

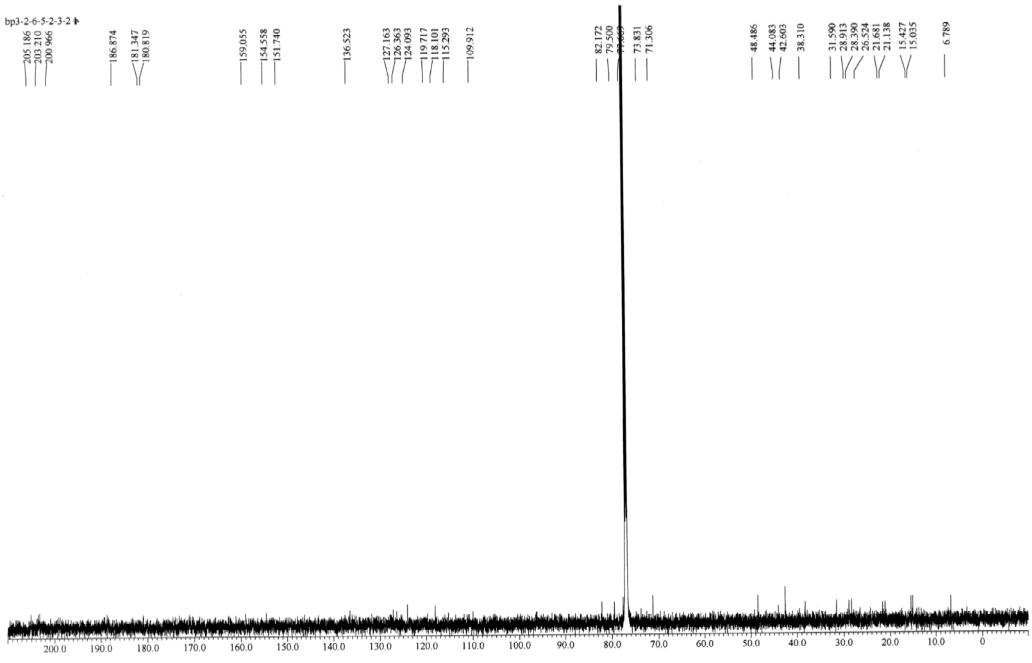

S32. ^{13}C NMR spectrum (150 MHz) of compound **4** in $CDCl_3$

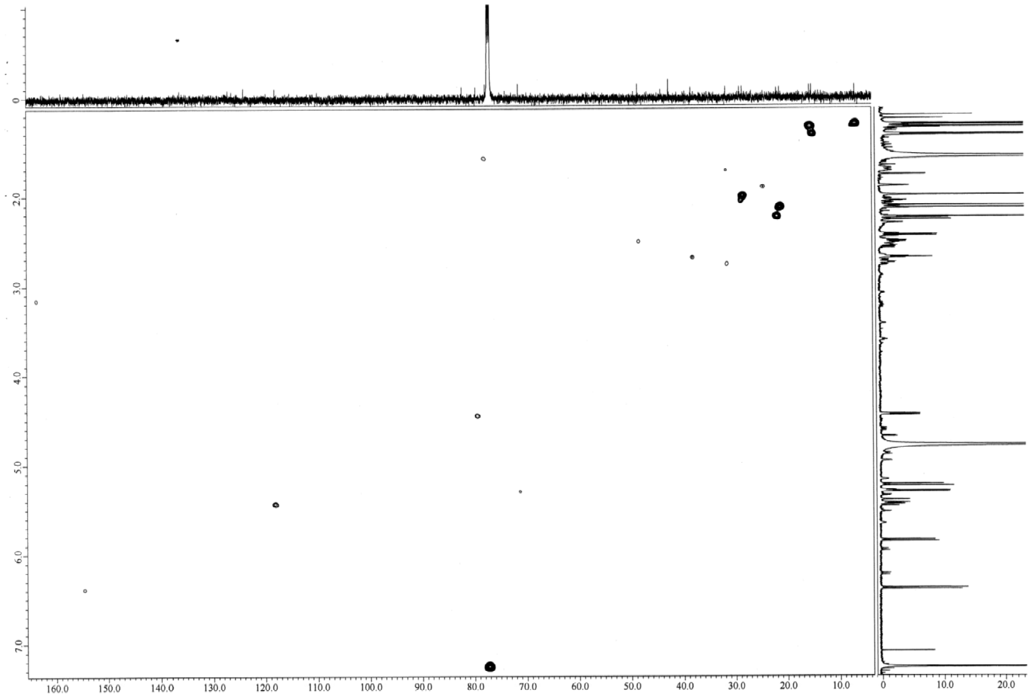

S33. HSQC spectrum of compound **4** in $CDCl_3$

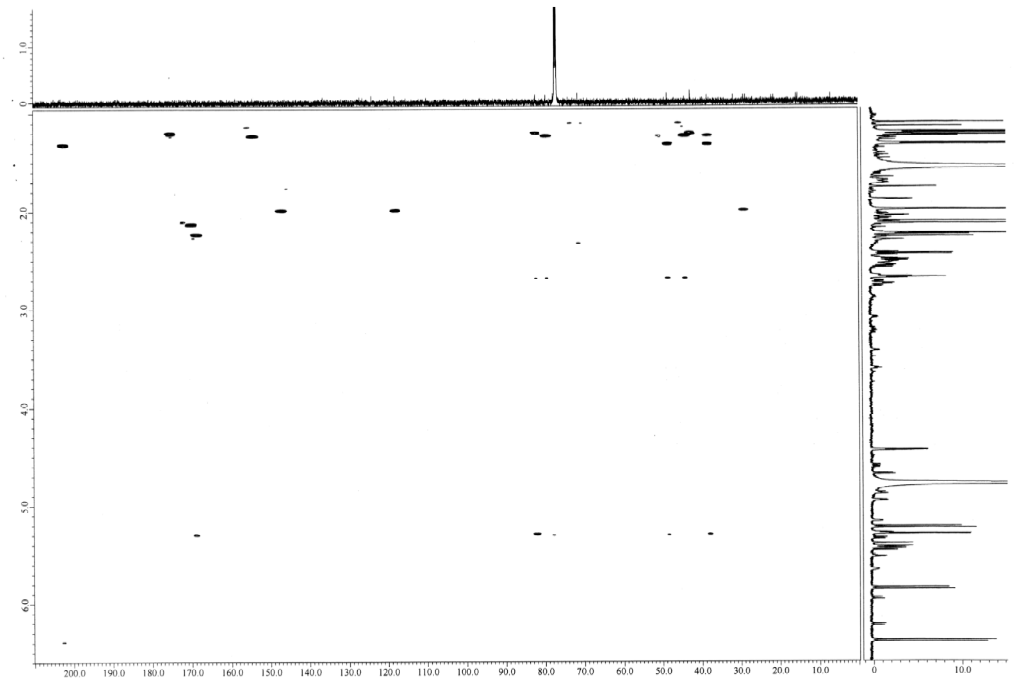

S34. HMBC spectrum of compound **4** in $CDCl_3$

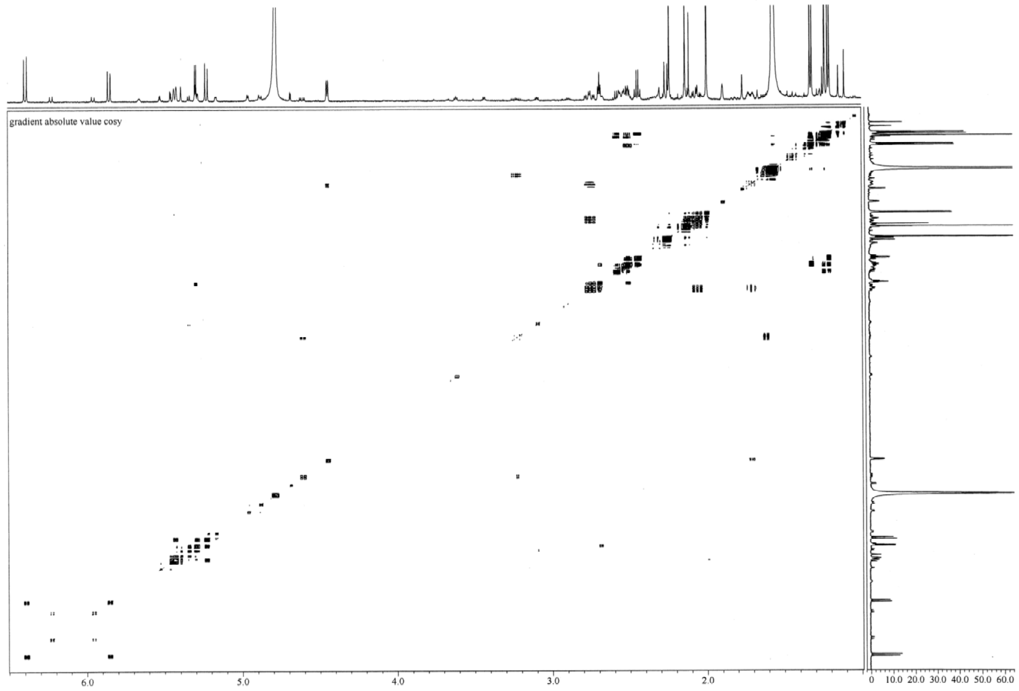

S35. ^{1}H-^{1}H COSY spectrum of compound **4** in $CDCl_3$

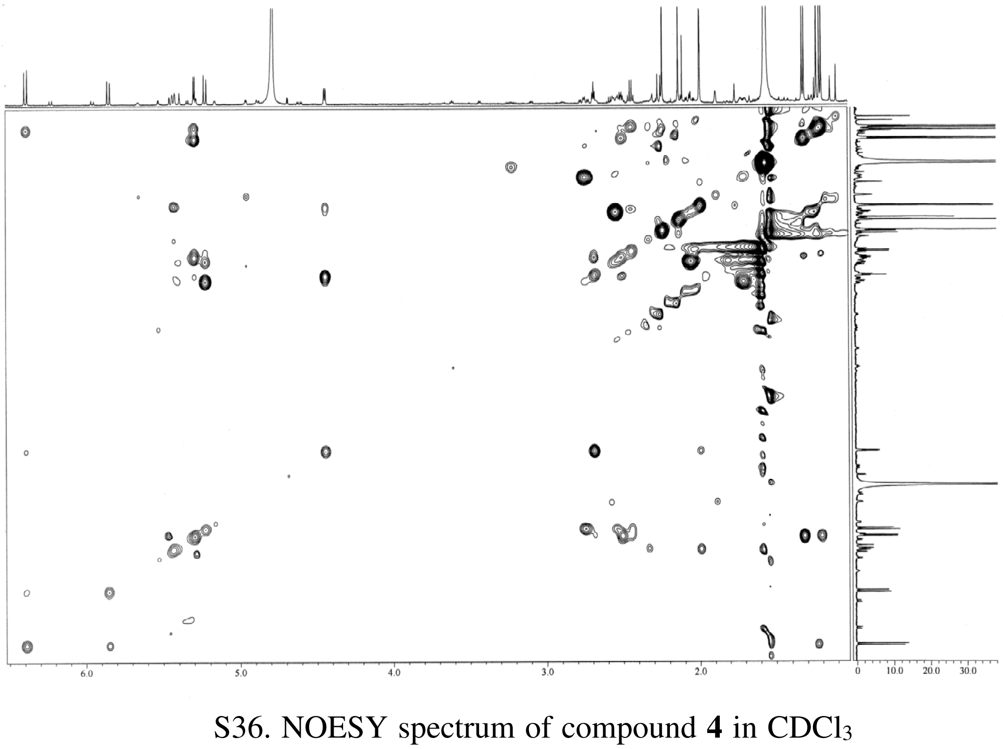

S36. NOESY spectrum of compound **4** in $CDCl_3$

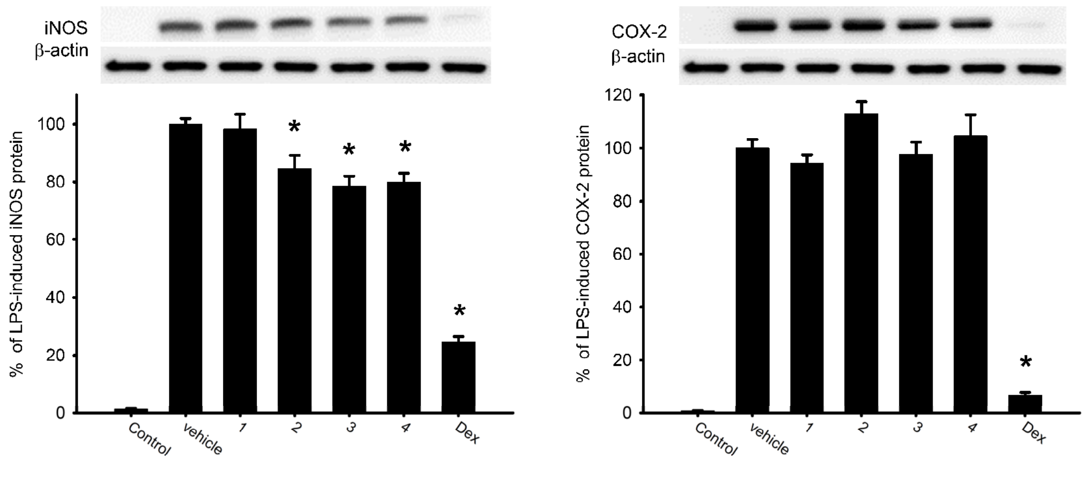

S37. Western blotting of **1–4**
